# Impact of genetic background and experimental reproducibility on identifying chemical compounds with robust longevity effects

**DOI:** 10.1038/ncomms14256

**Published:** 2017-02-21

**Authors:** Mark Lucanic, W. Todd Plummer, Esteban Chen, Jailynn Harke, Anna C. Foulger, Brian Onken, Anna L. Coleman-Hulbert, Kathleen J. Dumas, Suzhen Guo, Erik Johnson, Dipa Bhaumik, Jian Xue, Anna B. Crist, Michael P. Presley, Girish Harinath, Christine A. Sedore, Manish Chamoli, Shaunak Kamat, Michelle K. Chen, Suzanne Angeli, Christina Chang, John H. Willis, Daniel Edgar, Mary Anne Royal, Elizabeth A. Chao, Shobhna Patel, Theo Garrett, Carolina Ibanez-Ventoso, June Hope, Jason L Kish, Max Guo, Gordon J. Lithgow, Monica Driscoll, Patrick C. Phillips

**Affiliations:** 1 The Buck Institute for Research on Aging, 8001 Redwood Boulevard, Novato, California 94945, USA; 2 Nelson Biological Laboratories, Department of Molecular Biology and Biochemistry, Rutgers University, Piscataway, New Jersey 08854, USA; 3 Institute of Ecology and Evolution, University of Oregon, Eugene, Oregon 97403, USA; 4 Division of Aging Biology, National Institute on Aging, 7201 Wisconsin Avenue, Bethesda, Maryland 20892-9205, USA

## Abstract

Limiting the debilitating consequences of ageing is a major medical challenge of our time. Robust pharmacological interventions that promote healthy ageing across diverse genetic backgrounds may engage conserved longevity pathways. Here we report results from the *Caenorhabditis* Intervention Testing Program in assessing longevity variation across 22 *Caenorhabditis* strains spanning 3 species, using multiple replicates collected across three independent laboratories. Reproducibility between test sites is high, whereas individual trial reproducibility is relatively low. Of ten pro-longevity chemicals tested, six significantly extend lifespan in at least one strain. Three reported dietary restriction mimetics are mainly effective across *C. elegans* strains, indicating species and strain-specific responses. In contrast, the amyloid dye ThioflavinT is both potent and robust across the strains. Our results highlight promising pharmacological leads and demonstrate the importance of assessing lifespans of discrete cohorts across repeat studies to capture biological variation in the search for reproducible ageing interventions.

## Abstract (web summary)

Irreproducibility of biological findings is a major challenge for drug development. Here the authors examine the lifespans of 22 worm strains in three different laboratories and the effects of ten known chemicals to assess reproducibility in the face of variations in genetic background, chemical treatment and lab environment.

Considerable research effort is currently devoted to defining effective approaches towards healthspan extension, some with an emphasis on pharmacological interventions that prolong life. Success in this arena is likely to be derived from studies that target conserved pathways and exploit the complementary advantages of multiple model systems^1^,^2^,^3^,^4^,^5^. The *Caenorhabditis* genus contains remarkable genetic diversity^6^, exhibits relatively short lifespans and in general includes tractable model organisms. Collectively, these features allow for relatively inexpensive lifespan studies spanning diverse genetic backgrounds, which can be completed on a timescale of months. As importantly, *C. elegans* has long served as an important model system for understanding the biological basis of ageing, going back to the first discovery of genes responsible for extended longevity^7^,^8^,^9^,^10^. To address concerns about reproducibility and to identify compounds that extend lifespan in a robust manner, we created the *Caenorhabditis* Intervention Testing Program (CITP), a joint effort between the National Institutes of Health and three geographically separated research labs (Buck Institute for Research on Ageing, Rutgers University and the University of Oregon). Here our fundamental goal is to identify compounds that confer reproducible lifespan extension and health benefits to a genetically diverse panel of *Caenorhabditis* strains and species based on the idea that compounds with robust effects across a genetically heterogeneous population would have the highest probability of engaging conserved biochemical pathways that promote healthy ageing.

Reproducibility is considered a cornerstone of experimental science. At least one study has estimated the cost of flawed or irreproducible research at $28 billion per year in the United States alone, with 65% of this cost incurred by the pharmaceutical industry^11^. There are many reasons why studies may exhibit poor reproducibility. In molecular and cell biology, sources of variation include the quality and purity of reagents, uncontrollable daily fluctuations in microenvironment and idiosyncratic techniques of investigators^12^. When conducting animal experiments an even larger number of factors such as nutrients, genetic backgrounds and housing conditions may influence the observations^13^,^14^. Studies involving complex phenotypes such as ageing may be particularly sensitive to subtle alterations in such difficult to control factors. It has long been recognized that the lifespan phenotype exhibits stochasticity and therefore is highly variable across even closely related individuals from isogenic species^15^,^16^,^17^.

Experiments reporting the positive effects of drug-like molecules have in particular often failed in independent laboratories, a phenomenon recognized in the wider biomedical literature^18^,^19^,^20^,^21^. Despite these confounding phenomena, identifying pharmaceutical interventions that mitigate ageing and age-related chronic diseases is so potentially beneficial to society that the number of studies will, and should, increase dramatically in the future. To advance the goal of identifying pharmaceutical lead compounds, combat the variability of lifespan assays and establish rigorous testing methodology, the National Institute on Ageing assembled the Intervention Testing Program (ITP)^22^. Subsequently, this group has demonstrated effectively that multiple chemicals can increase the lifespan of genetically heterogenous mice^23^,^24^,^25^,^26^.

The CITP constitutes an independent, but complementary effort to the ITP, anticipated to allow a higher throughput analysis of candidate healthspan interventions. Invertebrates offer relatively rapid assessment of potential chemicals, thereby allowing for both higher throughput screening and greater sampling of genetic diversity. This is a key point, as it is likely to be critical to identify those compounds that robustly extend lifespan in multiple genetic backgrounds, to allow researchers to prioritize which chemicals to test in more complex organisms. Here we describe results from parallel replicate studies on the lifespans and life history traits of 22 *Caenorhabditis* natural isolates (strains), and further report the identification of several compounds that either promoted long life in a species specific manner or were generally effective across species.

## Results

### Developmental phenotypes and reproducibility

We chose 22 *Caenorhabditis* natural isolates spanning three species (*C. briggsae*, *C. elegans* and *C. tropicalis*) to maximize sampling of genetic and geographic diversity within each species^27^,^28^. In terms of total divergence, the genetic differences encompassed among these strains is comparable to sampling genomes from mice to humans^27^. We first tested whether the three independent labs could reproducibly score two major life history traits; the developmental time to reproductive adulthood (egg laid to adult deposition of the first egg), referred to as α-time and hermaphrodite self-fertility (total number of viable progeny). These studies also served to inform on the general health of the natural variant strains under the culture conditions we established to test compound-based ageing interventions.

**Figure 1:**
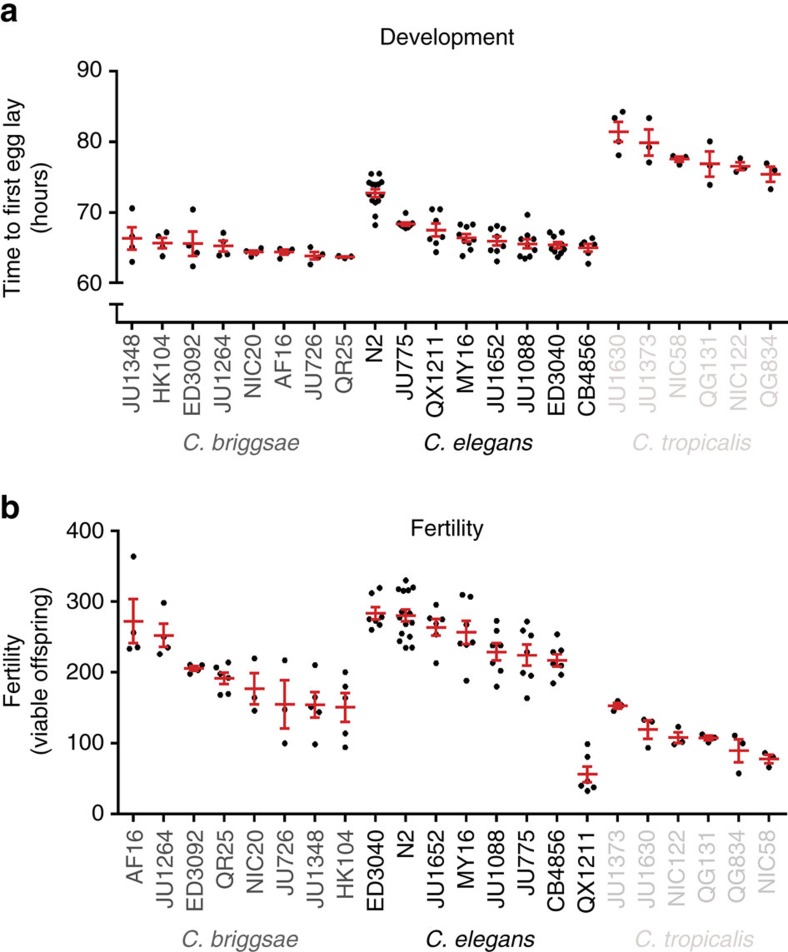
Summary of developmental time and fertility of 22 *Caenorhabditis* strains. (**a**,**b**) Graphical representation of the mean developmental time (**a**) and mean fertility (**b**) for 22 *Caenorhabditis* strains under the test culture conditions (see Methods). Each point represents the average of 20 individual animals, scored in one of the three CITP labs. Middle bar represents the mean with smaller bars indicating the s.e. Graphs are segregated by species such that eight *C. briggsae* strains are shown in grey, eight *C. elegans* strains are shown in black and six *C. tropicalis* strains are shown in off white. Statistical summaries of the parent data used to generate these graphs is presented in Table 1 and Supplementary Table 1, with the per-replicate estimates and sample sizes provided in Supplementary Data 1 and 2.

We determined the α-time for the 22 strains (>1,800 observations; see Supplementary Data 1 and 6), finding that the developmental rate at 20 °C is slightly delayed in *C. tropicalis* strains relative to *C. briggsae* and *C. elegans*, with good agreement among labs (Fig. 1a). Indeed, partitioning variation among potential sources of error using a general linear model (GLM) indicated that there was very little systematic difference among outcomes at the three laboratories (Table 1 and Supplementary Table 1), although there were some lab-specific differences among species and strains (roughly 4% of the total variance attributable to each source). The key finding was that the vast amount of variation (79%) was attributed to genetic variation among strains and species. These data suggest that growth conditions and practices for developmental time analysis were uniform across labs.

We also assayed hermaphrodite self-fertility under our culture conditions. Each of the species used here reproduces as self-fertilizing hermaphrodites and are limited by sperm production during development. The self-fertility of each strain was determined by counting the number of viable progeny born from individual animals. In general the *C. elegans* strains exhibited the highest fertility, with strain QX1211 the clear outlier (Fig. 1b). QX1211 exhibited lower fertility than any other strain and was observed to lay many eggs that failed to hatch (unquantified observations), indicating some level of embryonic lethality. As with the developmental rate results, we found that self-fertility scores were reproducible among labs (1% variance among labs), with slightly larger lab-specific differences among strains than observed for α-time (Table 1). Variation in fertility under our lab conditions can be assigned primarily to genetic background (63%) and ‘random' (unexplained) differences among individual animals (23%). Importantly, the data again support little systematic difference in scores (and therefore in culture conditions) across the three CITP laboratories.

### Some *Caenorhabditis* strain differences are due to genetics

**Figure 2:**
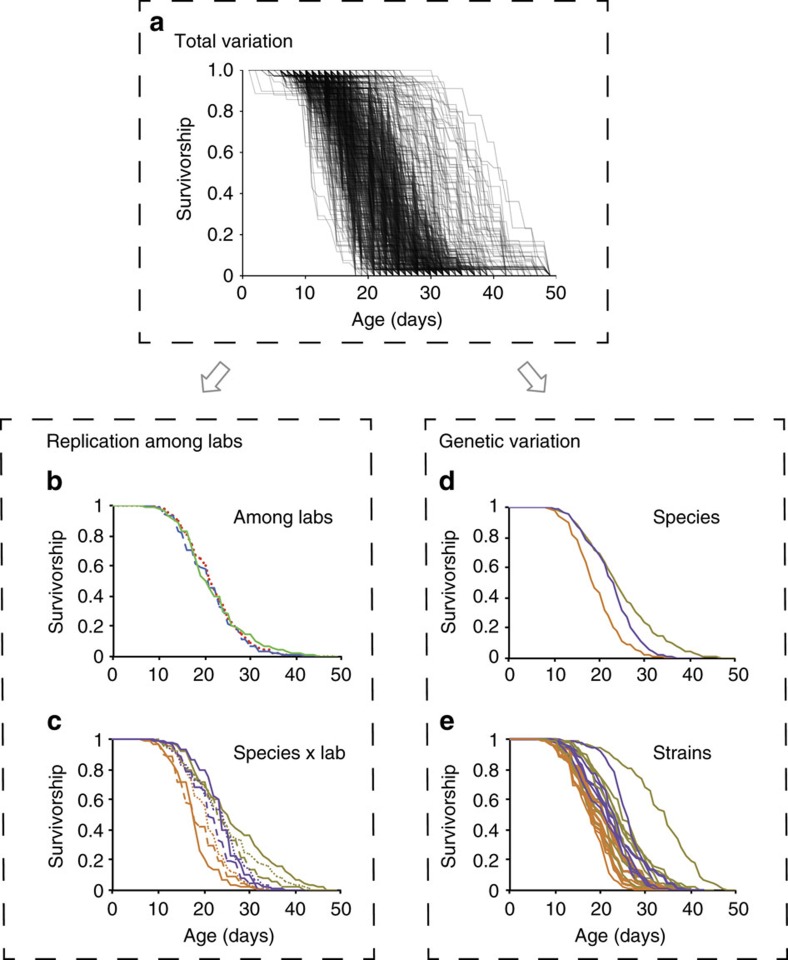
Natural and experimental variation in longevity among natural isolates of *Caenorhabditis*. (**a**) The set of survivorship curves displaying the total range of observed longevity for each of the 728 experimental replicates (plates) from three laboratories measured across the three species and 22 natural isolates measured in this study. The cause of plate-to-plate differences in responses can be attributed to different sources using a hierarchical analysis that partitions the total observed variation to known sources of genetic differences and replication error. Overall, the average longevity across the entire experiment did not differ across the three laboratories (**b**), although there were species- and strain-specific responses that varied from lab to lab (**c**). There were also distinct differences among species (**d**), but in fact more variation among strains within species (**e**). Relative percentages of the total variation attributable to each source are given in Table 2. Orange lines are *C. elegans*, tan are *C. briggsae* and purple are *C. tropicalis*. Dashed lines (blue) are replicates from the Buck Institute, solid lines (green) are from Oregon and dotted lines (red) are from Rutgers. Sample sizes and per-replicate estimates for means and medians are provided in Supplementary Data 3.

We cultured each of the 22 natural isolates under standardized conditions and scored survival, with each trial being initiated with ≥105 adults distributed over three technical replicate plates. All CITP sites collected data from three biological replicate trials (21,143 observations; experimental details in Methods section and Supplementary Data 3 and 8). We observed broad differences in longevity among test strains spanning a twofold range at 20 °C (Fig. 2). We found strong, repeatable differences in the average mortality dynamics among the three species, as well as among strains within species (Fig. 2). In general, both *C. briggsae* and *C. tropicalis* live longer than *C. elegans*. However, whereas both *C. briggsae* and *C. tropicalis* have reduced early life mortality relative to *C. elegans*, *C. briggsae* continues a pattern of reduced mortality throughout life; the mortality rate in *C. tropicalis* tends to increase late in life such that its maximum lifespan ends up being fairly comparable to *C. elegans*. Natural isolates within each species also differed from one another in terms of their lifespan, with differences in median lifespan generally ranging from 3 to 7 days within each species (Fig. 2 and Supplementary Fig. 1). The clear outlier is *C. briggsae* strain HK104, whose median lifespan is 30% longer than any other *Caenorhabditis* natural isolate in our analysis, including other *C. briggsae* strains. Genetic differences account for roughly 20% of the total variation in longevity observed (12% among species and 8% among strains within species; Table 2).

### Minimal among-lab variation in lifespan

Given that a critical component of the CITP plan is to reproduce findings among labs, we examined reproducibility of longevity data at three primary levels: variability in outcomes among labs, variability of outcomes among replicates within labs and sensitivity of outcomes to genetic variation within and among species. Partitioning variation in longevity to different potential sources of variation in a hierarchical manner using a GLM (see Methods), we determined that reproducibility for longevity measurements across the three laboratories is extremely high, with on average there being no differences at all among labs (Fig. 2, Table 2 and Supplementary Table 2). Although there were effectively no systematic differences among laboratories, there is some indication of heterogeneous, strain-specific differences among laboratories, with laboratory-specific species and laboratory-specific strain results, respectively, accounting for <1% and 7% of the total variation. As the variation among laboratories was minor relative to other sources of variation, we infer that our strict adherence to uniform procedures was successful in largely eliminating systematic differences in lifespan outcomes among labs.

### Replicate variation within each laboratory is relatively high

Interestingly, although we found that systematic differences among labs were minor, we calculated replicate-to-replicate variation within each lab to be relatively high. After accounting for other sources of variation, strong among-replicate differences remained, representing roughly 15% of the total variation in individual lifespan observed in our study (9% derived from the trial-specific effects, 6% from the among-plate in same trial differences and none from experimenter-specific differences). Thus, although the results obtained on any given day of a replicate trial tended to be fairly consistent with one another, conducting the same assay a month later could yield results as different as looking at a strain from a different species.

Given that we observe a relatively large amount of variation among trials across each of the three labs, despite strict adherence to standardized procedures and culture conditions, we conclude that a major challenge to reproducibility in this system may arise from trial-specific cohort responses to unidentified and apparently subtle differences in the assay environment, which vary similarly within each laboratory.

### Bimodal ageing for *C. briggsae* replicates

**Figure 3:**
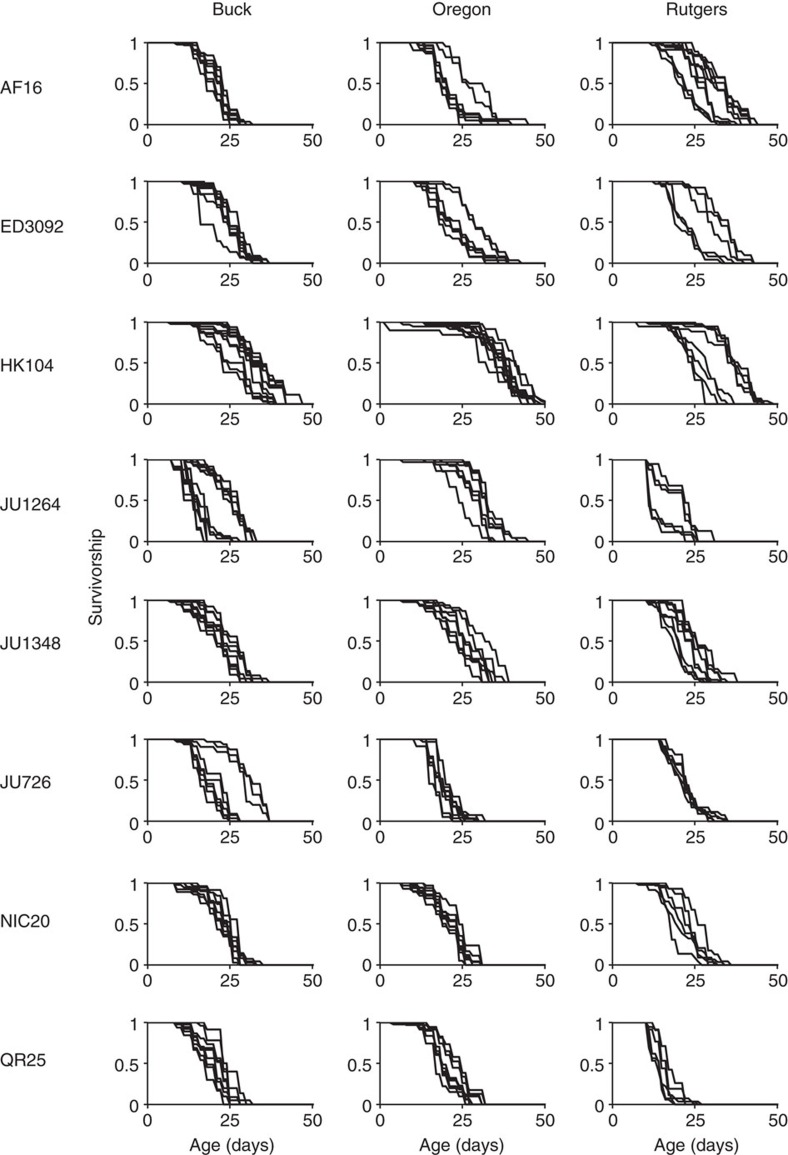
Variation in longevity within labs for each replicate plate for eight natural isolates of *C. briggsae*. It is noteworthy that in many cases a given natural isolate tends to display distinct patterns of responses under identical laboratory conditions rather than a continuous distribution of ‘error' among replicates. Among-replicate variation within each lab was a much larger barrier to reproducibility than variation in the average response of a strain across labs (Table 2). Each plate was initiated with *n*=35 animals.

The observed among-trial variation could simply be a random byproduct of tracking a phenotype (longevity) that is unlikely to be under tight regulatory control. Indeed, the large amount of residual variation in longevity (57%) is consistent with longevity being an inherently variable trait. However, the strikingly discrete nature of among-replicate variation for some lines, especially within *C. briggsae* strains, suggests that fundamental biology may underlie the observed trial-specific differences in lifespan outcomes (Fig. 3). For example, strain JU1264 exhibited distinct clusters of longevity trajectories across the different trials: cohorts either showed high early mortality or long life (Fig. 3). The absence of intermediate outcomes suggests that even populations reared under tightly controlled conditions may shift between discrete physiological states, perhaps induced by some unmeasured/unknown environmental factor. The propensity of a given strain to display distinct longevity trajectories varied from lab to lab, although all labs observed this phenomenon for one strain or another. *C. elegans* and *C. tropicalis* also displayed some degree of strain-specific differences of among-trial variation, although distinct differences in longevity trajectories are less obvious in these species than in *C. briggsae* (Supplementary Figs 2 and 3). If this state-shift is a general feature of this system, then reproducibility ‘error' at this level might actually reflect an inherent property of these species that cannot be eliminated without further knowledge of its root causes. At present, our controlled studies suggest that investigator, site, plate or reagent batch, overall temperature and humidity, and generational epigenetic factors associated with food availability are not factors in bimodal outcome; likewise, strains that generate males with highest frequency did not uniformly show this bimodal response. Our observations thus introduce an unexpected area for mechanistic investigation.

### Analysis of variation in intervention lifespan outcomes

Our initial characterizations identified a twofold range in median lifespan represented among the strains, which provides a strong substrate of diversity for testing generalized effects of compound interventions. To keep the experimental design logistically tractable for our initial test set of ten compounds, we focused on outcomes with three *C. elegans* strains and three *C. briggsae* strains that had fared well under lab growth conditions (46,231 observations; experimental details in Methods and Supplementary Data 4 and 9). The strains we selected captured the range of longevities that we had observed in our initial broad survey. For the first intervention studies we selected ten chemicals that had either attracted particular interest in the ageing field (aspirin^29^ and resveratrol^21^), were previously reported to extend the lifespan of the *C. elegans* strain N2 (α-ketoglutarate (α-KG)^30^, curcumin^31^, α-lipoic acid (α-LA)^32^, propyl gallate (PG)^32^, quercetin^33^,^34^ and valproic acid (VA)^35^) and/or appeared particularly robust when given to N2 in our own laboratories ((NP1)^36^ and ThioflavinT (ThT)^31^).

Another consideration for the initial test set was that the compounds had been predicted to influence ageing by a range of primary mechanisms. For example, ThT promotes protein homeostasis^31^ and VA extends lifespan via activation of the transcription factor DAF-16 (ref. 35), whereas NP1, α-KG and resveratrol had all been reported to act as dietary restriction (DR) mimetics. To begin to assess variability in lifespan assays involving chemical treatments, we first tested a single concentration of each compound. In general, we chose concentrations previously reported to extend median lifespan in the *C. elegans* N2 strain.

We identified a similar pattern of variation in the compound trials as we reported above for the baseline, non-intervention studies (Table 2 and Supplementary Tables 2–3). That is to say, partitioning variation among potential sources of error using a GLM again established that little of the observed variation was attributed to differences among labs and minor variation due to combinations of lab-species, lab-strain and lab-compound effects (0.1–0.5% of total variation attributed to each). In contrast, we attributed 9.7% of the total variation to reproducibility within each lab. For interventions however, this among-trial variation was primarily associated with plate-to-plate differences (possibly due to application of the compounds to the plates, which is executed on a per plate basis). Genetic differences and individual differences among animals accounted for most of the variation in the intervention studies (∼44% each; Table 2). Genetic differences were proportionally more important for the compound interventions than in the baseline assays, probably because we intentionally sampled strains covering a wide range of baseline longevities and, as will be seen, treatment by many compounds tends to heighten differences among strains. Overall, then, although the three labs were able to create a high degree of reproducibility across all longevity assays, most replication ‘error' appears rooted in trial-to-trial differences that impact the three lab outcomes similarly. Collectively, our results indicate that for both chemical and baseline lifespan assays, it is critical to assess the lifespans of discrete cohorts in repeat studies, to capture the expected biological variation.

### ThT extended lifespan in a broad range of strains

**Figure 4:**
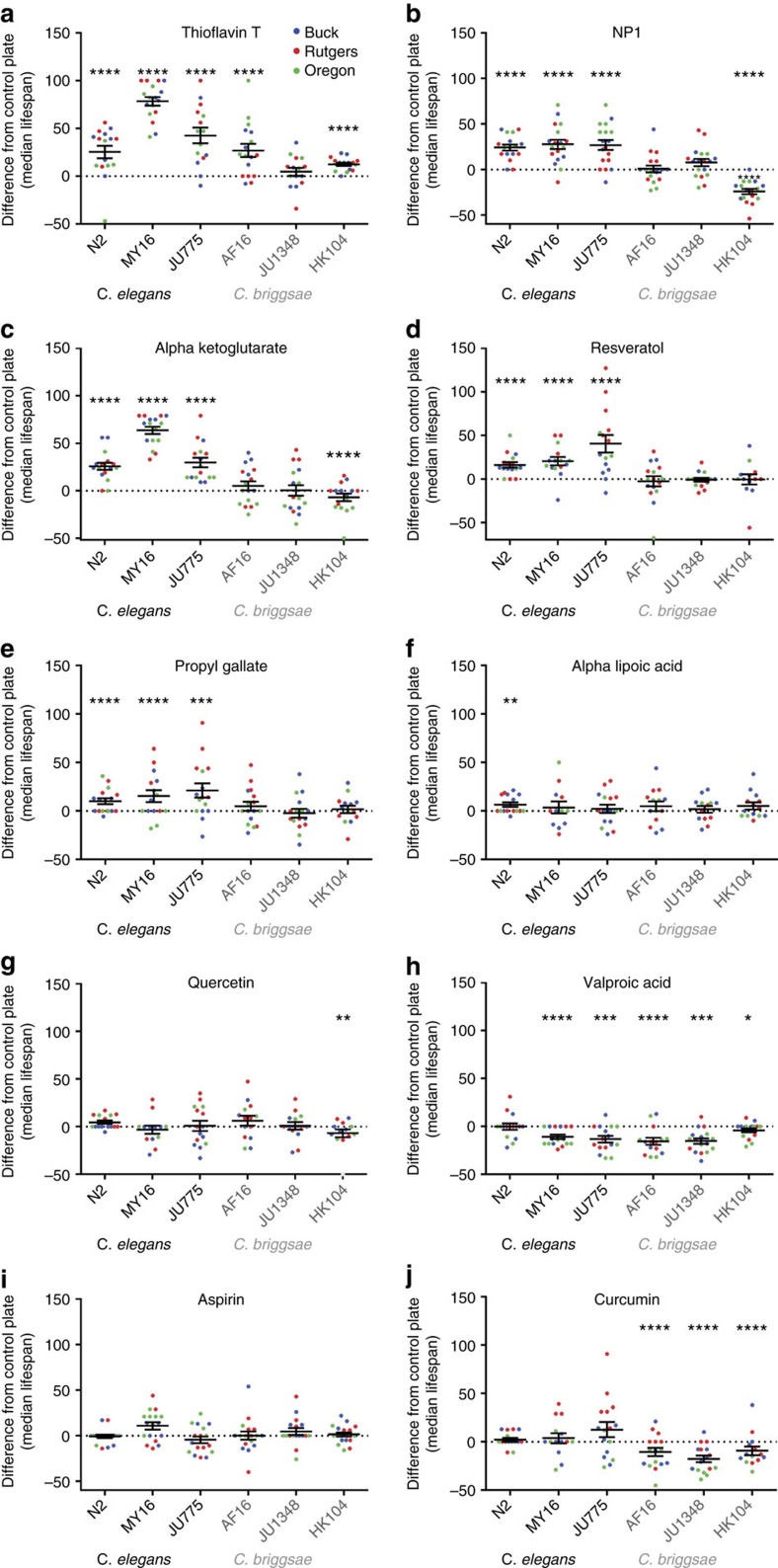
Chemical effect on the median lifespan of six *Caenorhabditis* strains. (**a**–**j**) The effect on median lifespan from ten different chemical treatments is shown for six *Caenorhabditis* strains. The six strains consist of three *C. elegans* species (N2, MY16 and JU775, black text) and three *C. briggsae* species (JU1348, AF16 and HK104, grey). The per cent difference in median lifespan was determined by calculating the median lifespan for each plate population (single plate lifespan assays starting with 35–40 animals, each site at least 6 plates in at least 2 biological replicates). Every chemical test plate had a control plate associated with it (diluent only control plate, that was maintained with the test plate), which contained animals from the same egg lay and was always scored by the same technician as the test plate. In all graphs each point represents the percent difference in median lifespan between two single plate populations, one containing the chemical being tested and the other containing the diluent control. Data incorporate censored animals in calculating median lifespan. Points are colour coded to indicate the lab the data was collected in, as indicated in key on **a**. Middle bar represents the mean with small bars, indicating the s.e. Asterisks represent *P*-values from the CPH model (Supplementary Table 4; ****P<0.0001, ***P<0.001, **P<0.01 and *P<0.05). Summaries from the statistical analysis of the parent data used to generate these graphs are included in Table 2 and Supplementary Tables 2 and 4. Sample sizes and per-replicate estimates for means and medians are provided in Supplementary Data 4.

Having addressed the overall reproducibility in our experiments, we next examined compound-specific effects on lifespan extension. We identified several compounds that had positive effects on lifespan. However, depending on the compound, these effects appeared to be differentially influenced by genetic background (Supplementary Fig. 4). ThT was the most robust of the chemicals we tested, as it significantly and reproducibly extended lifespan in five of the six strains tested, with only JU1348 failing to respond to treatment with significant lifespan extension (Fig. 4a, Supplementary Fig. 5 and Supplementary Table 4). In addition to being the most robust of the treatments, ThT also showed the most potent effect. We found that in some trials, for certain strains, ThT-treated populations exhibited a doubling of the median lifespan relative to control-treated populations (Fig. 4a and Supplementary Fig. 5). The average median lifespan (across all the trials for a given strain) ranged from a small but reproducible effect on HK104, the long-lived *C. briggsae* strain, to a significantly large effect (70% extension of median lifespan) on *C. elegans* strain MY16 (Fig. 4a). The potent and robust longevity promoting effect that ThT exhibited on these *Caenorhabditis* strains suggests that the molecular mechanism targeted by ThT is a major determinant of lifespan that is conserved across divergent genetic backgrounds.

### DR mimetics exhibited similar strain specific responses

NP1 is a synthetic compound, which we previously identified as promoting lifespan in the N2 strain through a DR mechanism^36^. In this study, we found that NP1 exhibited species- and strain-specific effects on lifespan (Fig. 4b and Supplementary Fig. 6). The *C. elegans* strains all showed significantly extended lifespan when treated with NP1, relative to control-treated animals (Fig. 4b, Supplementary Fig. 6 and Supplementary Table 4). On average, NP1 caused a remarkably consistent and fairly potent effect on lifespan across these strains (∼30% longer median lifespan relative to control treated populations), with the most reproducible effects (among the strain specific replicates) observed in the N2 strain and the most potent effects (across all strain replicates) observed in the wild isolated strains (MY16 and JU775; Fig. 4b). The effect of NP1 on the lifespan of the *C. briggsae* strains was more varied. NP1 did not have a significant effect on lifespan in either AF16 or JU1348, but it consistently and fairly potently shortened the lifespan of strain HK104 (∼30% shorter median lifespan on average compared with control-treated populations; Fig. 4b, Supplementary Figs 4 and 6, and Supplementary Table 4).

Similar to the effects observed from NP1 treatment, the metabolite α-KG, which was also previously implicated in DR^30^, significantly extended the lifespan of all the *C. elegans* strains tested and did not have a significant effect on the lifespan of *C. briggsae* strains AF16 and JU1348, but shortened the lifespan of strain HK104 (Fig. 4c, Supplementary Figs 4 and 7, and Supplementary Table 4). Although the trends observed were the same for both chemicals, the magnitude of the effect on lifespan from treatment with these chemicals was distinctly different in two of the strains. αKG potently extended the lifespan of the *C. elegans* strain MY16, with treated populations commonly living 60% longer than control treated animals, whereas its negative effect on the lifespan of strain HK104 was much weaker and less consistent than was observed for NP1 (Fig. 4c, Supplementary Figs 4–7 and Supplementary Table 4). Resveratrol has also been reported to promote DR (Fig. 4d and Supplementary Fig. 8)^21^. Although there has been considerable controversy surrounding resveratrol and its proposed mechanism of action, most investigators studying resveratrol effects on *C. elegans* lifespan have documented small but significant beneficial effects on the laboratory standard N2 strain^19^,^37^. We also observed this effect and further found that similar to NP1 and α-KG, resveratrol also significantly extended the lifespan of the other *C. elegans* strains tested. However, unlike NP1 and α-KG, we did not observe any overall significant lifespan effect on any of the *C. briggsae* strains treated with resveratrol compared to control treated populations (Fig. 4d, Supplementary Figs 4 and 8, and Supplementary Table 4). Overall, our work with candidate DR mimetics suggests that there are promising benefits from such pharmacological interventions, but at the same time our data seem to indicate that these chemicals tend to exhibit highly variable outcomes, dependent on the genetic background of the treated subjects.

### Compound that did not reproduce previous longevity outcomes

In previous studies, three compounds that can exhibit antioxidant properties (PG, α-LA and quercetin) extended the lifespan of the standard laboratory strain N2 (refs 32, 33). We found that treatment with PG significantly, but relatively weakly, extended the lifespan of diverse *C. elegans* strains relative to control-treated populations. However, we did not observe significant effects on the longevity of the *C. briggsae* strains from treatment with PG (Fig. 4e and Supplementary Table 4). Compared with the other treatments we assessed, this profile appears most similar to the pattern observed in the resveratrol treatments. α-LA-treated populations of the N2 strain exhibited a small but significant lifespan extension relative to control-treated populations. However, none of the wild strains exhibited a significant lifespan response to α-LA (Fig. 4f and Supplementary Table 4). Quercetin did not significantly extend the lifespan of any strain tested (Fig. 4g and Supplementary Table 4). Overall, there was little evidence that compounds with antioxidant properties extended lifespan using the test conditions described here, which might reflect the *in vivo* pleiotropy of reactive oxygen species, which have been shown to act in pro-longevity signalling cascades, in addition to exhibiting well-known negative impacts on cellular systems^38^.

In our study, anticonvulsant drug VA (Fig. 4h) failed to extend lifespan in any strain, an initial surprise, because VA has been previously documented to extend lifespan of *C. elegans* strain N2 in multiple replicate experiments^35^. Comparison of the CITP treatment strategy with the published report suggests that differences in outcomes may be due to use of distinctly different treatment protocols (for example, we treated only during adulthood, whereas Evason *et al*.^35^ treated the animals from conception). Similarly, under our test conditions, aspirin and curcumin (Fig. 4i,j) did not exert robust positive changes on longevity across the test set. These differences from previously published results may also reflect differences in test conditions.

### Species-specific dosage responses

**Figure 5:**
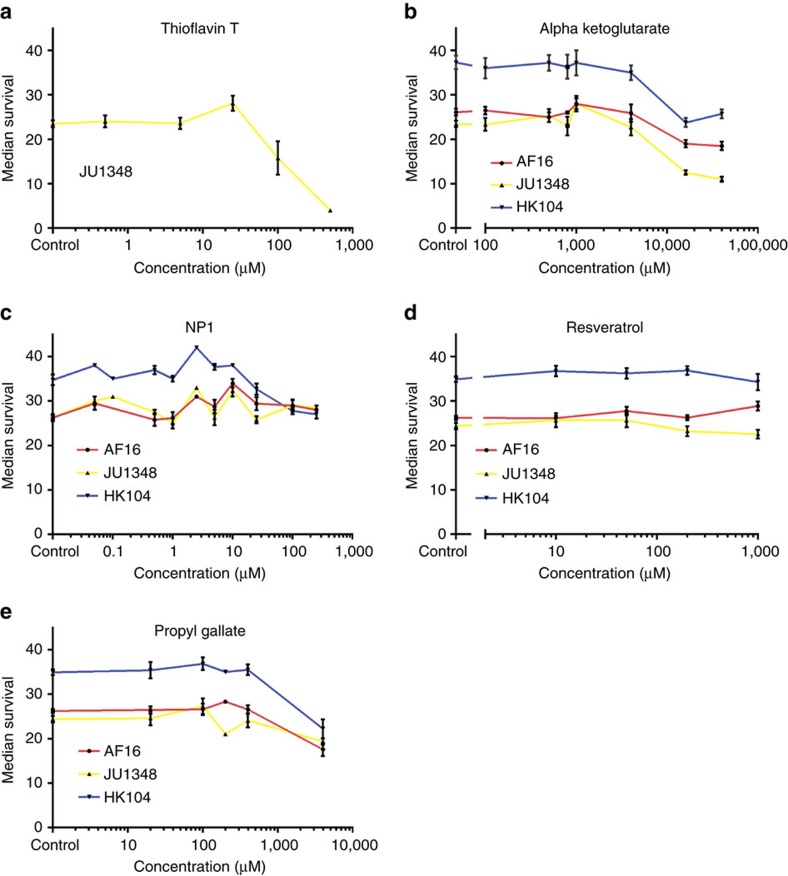
Dosage effects on the lifespan *C. briggsae* with select positive chemicals. (**a**–**c**) Dose response effects on the median lifespan of select *C. briggsae* strains after treatment with chemicals that exhibited strong positive effects on the *C. elegans* strains. Dosing was performed only on strains that failed to respond positively in the initial tests (single dose experiments), as we did not attempt to identify peak responses, instead we only sought to identify whether positive effects could be obtained by altering doses. Chemical doses were chosen to center around the effective dose identified for *C. elegans* strains and were sometimes expanded after preliminary rounds of testing. ThT exhibited a positive effect on strain JU1348 at 25 μM, but was profoundly toxic to all strains at and above 100 μM (**a**). NP1 exhibited a positive effect on AF16 and JU1348 at 10 μM relative to control treated populations and showed reduced toxicity to HK104 at low micromolar concentrations (**b**). αKG did not exhibit clear positive effects in any of the *C. briggsae* strains tested, but exhibited toxicity at high millimolar concentrations (**c**). None of the resveratrol doses assayed appeared to alter the median lifespan of any of the *C. briggsae* strains tested (**d**). PG showed a negative effect on median lifespan for all of the *C. briggsae* strains tested at low millimolar concentrations (**e**). Median lifespans were determined from single plate populations. Mean values are plotted here with small bars, indicating the s.e. (sample size and statistical summaries are included in Supplementary Data 5).

The most robust compounds from our primary test were quite potent in the tested *C. elegans* strains, yet other than ThT, these same compounds essentially failed to exert positive effects in the *C. briggsae* strains. One possibility we considered is that *C. briggsae* may exhibit distinctly different dosage sensitivity than *C. elegans*. This hypothesis seemed reasonable as the dosages tested were chosen based on published reports of effectiveness in the *C. elegans* strain N2. To further explore the chemical responsiveness of the *C. briggsae* strains that failed to respond, we tested a range of doses for the chemicals that were identified as positive in our initial tests. Dose–response experiments with ThT indicated that in fact ThT can have a positive impact on JU1348 but only at lower dosages than we originally examined (Fig. 5a). As in the other strains, the effective dose of ThT is relatively close to the minimum toxic dose (see Discussion). In general, ThT was found to be effective at extending lifespan across all genetic backgrounds tested.

In contrast, the reported DR mimetics (NP1, αKG and resveratrol), which each showed positive effects across the *C. elegans* strains, resulted in distinct responses from the *briggsae* species (Fig. 5b–d). For αKG and resveratrol, the three *C. briggsae* strains all showed similar overall dose-dependent responses. The *C. briggsae* strains treated with αKG did not clearly respond with lifespan extension at doses in the micromolar range but did exhibit shortened lifespan in the mid-millimolar range (Fig. 5b). In contrast, we did not detect any clear response from the *C. briggsae* strains treated with resveratrol (Fig. 5d). This lack of response may indicate that the chemical is unavailable to the animals, has low toxicity or that our dose range was too low to observe an effect. The upper limit of our dose testing for resveratrol was 1 mM, an order of magnitude higher than the dose that exhibited positive effects on the *C. elegans* strains. The *C. briggsae* strains treated with NP1 displayed more variable responses. Both AF16 and JU1348 showed some indication of a positive effect on lifespan from treatments with NP1 in the low micromolar range, whereas in this same range HK104 exhibited negative effects. The shortening of lifespan effect from NP1 became more pronounced at mid- to high-micromolar concentrations of NP1. We did not identify any dose of NP1 that clearly extended the lifespan of HK104. We also tested PG, which showed positive effects on the *C. elegans* strains, for dose-dependent effects on the *C. briggsae* strains. PG showed a similar profile as αKG, with no clear effect at low doses and negative effects observed at low millimolar levels (Fig. 5e). Collectively, these results suggest that the *C. briggsae* strains exhibit different dosage specificities than *C. elegans* and further that some of these chemicals have conserved effects, while others show species-specific and strain-specific effects.

## Discussion

Coupling the general challenge of reproducibility in ageing studies with the goal of promoting healthy ageing, the National Institute on Ageing created the ITP^39^. This programme has been testing potential pro-longevity interventions in selected hybrid mice in three participating laboratories. ITP efforts have been successful in showing that it is possible to identify compounds that increase mouse lifespan in independent laboratories and have generally encouraged research into pro-longevity compounds^24^,^40^. We established a similar experimental platform for *Caenorhabditis* nematodes, to accelerate the discovery of compounds that robustly extend lifespan, while simultaneously examining the effects of variability in genetic background by testing compounds across multiple natural isolates drawn from three genetically divergent species. Using this platform, we have uncovered novel features of ageing in *Caenorhabditis* and a range of responses to compounds including species-specific trends, strain-specific effects and variable responses. Given that the strict coordination of experimental protocols among our labs limited between-lab variability, our findings highlight the challenges of scientific reproducibility with complex phenotypes such as longevity. Overall, we find that for both natural ageing and ageing with pharmacological interventions, repeated lifespan trials across three labs under tightly controlled experimental conditions yielded similar outcomes. At the same time, our results highlight that factors such as trial-to-trial variation and inherently variable responses to interventions that may be engrained in basic biology complicate efforts towards precise reproducibility.

At the outset of this project we noted extensive differences in details of media preparation, bacterial growth, animal handling and experimental protocols when we compared working practices among CITP labs. Although a fully ‘robust' intervention might withstand myriad protocol differences, we recognized that interpretation of mixed or negative results would be clarified only if we conducted experiments as similarly as possible. By eliminating most systemic sources of variation, we anticipated that we could focus on genetic diversity and other sources of variability during data analysis. Consideration of the numbers of reagents, protocols and human behaviour involved led to the appreciation of the complexity of options for executing any given experiment. As precise replication and adherence to protocol is likely to be required, to fully reproduce studies across labs, we defined a set of experimental protocols that were rigorously followed at all sites^41^. Although we were able to successfully eliminate among-lab variation in outcomes for the CITP, we did not systematically test each variable in our studies for impact on variation. We do not therefore suggest that the protocols we followed were the best possible practices; instead, we focused on strict execution of similar experimental details at all sites. Importantly, our documentation of relatively high variability in lifespan results across biological replicates independent of the lab conducting the experiment should be taken into account when one assesses the effects of chemicals or genetic backgrounds on lifespan. Our study indicates that even when following the same methods, insufficient replication of trials could account for failures to reproduce previous studies.

Our focus on rigorously adhering to defined methods to reduce variability between sites necessitated making choices about specific methodologies for which there was no standard across the field. In particular, these related to the use and dosage of FUdR, use of live bacteria and the method of chemical delivery, all of which have previously been found to be confounding factors in lifespan studies^42^,^43^,^44^,^45^,^46^. We specifically chose to use relatively low doses of FUdR, live bacteria cultures, added chemicals directly to culture plates only after bacteria had grown to saturation and only exposed adult animals to chemicals. These methods were mainly chosen to promote throughput and help mitigate possible effects of chemicals on bacterial growth and density. We also note that rigorous adherence to specific methodologies across a scientific field could limit serendipitous findings related to varying those conditions. In this study, it is possible that these selections resulted in discrepancies of methodology between ours and previously published studies. These differences may have contributed to our failure to reproduce several previously reported effects. Specifically, we did not observe previously reported lifespan extension of the standard laboratory strain N2 when treated with quercetin, VA, aspirin or curcumin^29^,^31^,^33^,^35^. Further investigation would be required to determine whether procedural differences are responsible for a given failure to replicate previous studies and/or whether these particular compounds are particularly sensitive to specific experimental circumstances.

The comparative life history analysis of our 22 wild strains revealed interesting features. We found that both α-time and fertility were remarkably similar for organisms collected over a wide geographical area. This consistency may indicate that selective pressure for fast growth and high fertility is shared across the strains. We found the variation for adult survival to be much higher than variation for self-fertility. Increased variation in the longevity measure within the *Caenorhabditis* genus is consistent with the idea that normal ageing processes are not under selective pressure and therefore more likely to drift randomly.

We found that multiple *C. briggsae* strains exhibited a striking bimodal survival outcome in all three labs. For example, a total of 25 survival assays were undertaken on HK104 across the three labs. We observed that 13 cohorts were relatively short-lived and 12 cohorts were long-lived. We observed this bimodality in almost all *C. briggsae* strains to some degree. The bimodal pattern appears to be a systematic source of within-lab variance intrinsic to the system. Strong evidence supports that even single-celled organisms can adopt distinct physiological states^47^. Our findings raise the question as to whether *C. briggsae* might have the capacity to transit through distinct physiological states according to unidentified cues. The *Caenorhabditis* capacity to adopt distinct physiological states could also account for some of the reported heterogeneity in behaviours and phenotypes.

A core premise of the CITP is that compounds that extend lifespan across genetically diverse organisms are strong candidates for further testing and mechanistic dissection in multiple models, and eventually humans. Indeed, among a small initial compound test set, we found that ThT extended lifespan in all of the six strains tested. ThT is a well-known laboratory reagent, commonly used as a histological stain for protein aggregates and in particular amyloids. ThT has previously been shown to suppress the aggregation and toxicity associated with the expression of a human neurotoxic peptide (Aβ_3-41_) and promotes protein homeostasis in a range of protein homeostasis models in *C. elegans* strain N2. Furthermore, the *in vivo* effect on lifespan has been associated with a wide range of gene expression changes impacting proteostatic functions including molecular chaperones, autophagy and the proteasome^31^. Collectively, these findings have been interpreted as indicating that ThT promotes lifespan by improving protein homeostasis^31^. Whether or not enhanced protein homeostasis is indeed the operative mechanism causative for the ThT effect on lifespan extension, our results suggest that ThT acts through a general longevity mechanism conserved across highly divergent *Caenorhabditis* strains, worthy of focused attention for intervention strategies. However, it seems that such research will ultimately need to be directed towards identifying less toxic derivatives of ThT or structurally unique compounds that target the same pathways as ThT, as we observed toxicity from ThT in addition to robust and profound lifespan extension.

Our enthusiasm for ThT as a direct candidate for pre-clinical studies was somewhat diminished by observations of this hormetic dose response (Fig. 5a). Among all the chemicals tested for dose responses in *C. briggsae*, ThT was the most toxic, killing off JU1348 rapidly when administered in the mid-micromolar range (Fig. 5). Interestingly, we found that the low micromolar doses at which ThT was effective in promoting lifespan was quite close to the dose at which it was toxic (Fig. 5a). We had previously observed a similar dose response profile for the lab adapted N2 strain^31^ and this phenomenon also fits in well with our observations that cohorts treated with ThT sometimes suffered high early life mortality with the survivors exhibiting low mortality in late life (Supplementary Figs 4 and 5). Despite these observations, we do not favour a mechanistic model of ThT activity as strictly hormetic (beneficial effect arising from low-dose administration of a toxin). It is important to note that, although low doses of toxins can sometimes have beneficial effects on lifespan through unclear and perhaps myriad mechanisms (including upregulation of stress response pathways), this is not always the case^48^,^49^,^50^,^51^. The magnitude of the ThT effect on lifespan appears to exceed previously reported examples of such chemically induced phenomenon in *C. elegans*. However, without an understanding of the molecular targets we cannot rule out indirect upregulation of stress response pathways as causative for the observed ThT lifespan extension. It will be interesting to determine whether the lifespan extending effects and toxicity of ThT can be separated. If the two phenomena are found to be inseparably linked, perhaps future research can identify less potent compounds that target the same pathways, such that even at high doses they only reach the effects of the low micromolar ThT treatments.

In our initial experiments we found that several positive acting chemicals (NP1, αKG, resveratrol and PG) exhibited positive lifespan effects on the *C. elegans* strains, but not on the *C. briggsae* strains, revealing species-specific outcomes. Our dose–response assays with these chemicals suggest that in fact some of these chemicals can be effective in the *C. briggsae* strains, albeit at lower doses (Fig. 5). This result indicates that although species-specific interactions are apparently present, a major issue for research comparing chemical treatments across species is identifying the different dosing requirements that arise from distinct chemical sensitivities. Layering dosage-specific effects on top of the demands of replication across multiple genetic backgrounds rapidly increases the scale of studies needed to examine the effectiveness of individual compounds, yet this is a core component of the recent push towards personalized or precision medicine^52^.

It is noteworthy that treatment with two chemicals, reported to activate DR (NP1 and αKG), resulted in strikingly similar strain response profiles, including extended lifespan in the *C. elegans* strains but shortened lifespan in the *C. briggsae* strain HK104 (Fig. 4b,c). This phenomena might shed light on a similar phenomenon observed in diverse mouse strains, in which lifespan can be either extended or reduced by DR, and underscores that metabolic interventions that engage DR might be particularly sensitive to genetic background^53^. HK104 might have its native metabolism set close to or in DR, such that the NP1 intervention pushes the strain closer to the starvation/deleterious level of DR^54^. Alternatively, distinct bioavailability properties in these strains might underlie this differential outcome (for example, HK104 may accumulate NP1 to toxic levels). Although more DR mimetic interventions need to be tested before strong conclusions can be drawn, our observations suggest that particular intervention response profiles across the CITP strain test set may eventually be informative on mechanisms of compound action.

Here we have described a multi-site *Caenorhabditis* longevity study conducted with attention to tightly replicating experimental conditions, an approach that enabled us to define sources of variation in survival analyses that provided a powerful assessment of our experimental reproducibility. We documented the presence of relatively high variability (∼10%) in lifespan results among biological replicates (trials) and demonstrate that this was independent of the lab, person or the site running the experiment. This demonstrated that previously anecdotal observations of variation in lifespan outcomes in fact occurs and exists for multiple species of the *Caenorhabditis* genre, even under tightly controlled experimental conditions. Such variation may be a general characteristic of ageing studies across species and highlights the importance of conducting highly replicated experiments, which is a core element of the CITP approach. Another major feature of the CITP is the use of genetic variation within and between species as a probe for examining the robustness of longevity-enhancing effects of specific compounds. Indeed, we find genetic variation to be very important for some compounds, whereas other compounds are robust across labs, replicates and genetic backgrounds. Given our collective long-term goal of identifying interventions effective in improving human health and longevity, these latter types of compounds seem particularly promising leads for further testing in vertebrates.

## Methods

### Organism cultures and strains

*Caenorhabditis* cultures were grown and maintained essentially as previously described^55^. All worm cultures were maintained at 20 °C with 80% humidity in Biological Incubator Model I-36NL (Percival Scientific). We used an agar concentration of 23 g l^−1^ in nematode growth media (NGM) plates to reduce worm burrowing. The streptomycin-resistant bacteria strain OP50-1 was used as the nematode food source. Streptomycin was only used in bacteria-specific cultures and not in NGM plates.

Detailed standard operating procedures and the same reagents were used at each site to ensure uniformity of culture practices^41^. Standard operating procedures covered (among other things) plate and drug preparations, periodic thaw schedules of fresh organism stocks (from frozen stocks prepared and distributed from a single lab) and the disallowance (from assays) of worm populations that had experienced starvation, contamination or thawing within three generations. Technicians were not blinded to the strains and conditions during any of the experiments reported in this manuscript.

The 22 nematode strains were selected from three hermaphroditic species of *Caenorhabditis*: *C. elegans* (CB4856, ED3040, JU1088, JU1652, JU775, MY16, N2 and QX1211), *C. briggsae* (AF16, ED3092, HK104, JU1264, JU1348, JU726, NIC20 and QR25) and *C. tropicalis* (JU1373, JU1630, NIC122, NIC58, QG131 and QG834).

### Developmental time assay

The average developmental time for each strain was determined by scoring the time interval between when an egg was laid, until its development into an egg laying adult (α-time). Specifically, 1 h egg lays were performed with 50 first-day adults. Twenty to 25 of the resulting eggs were separated onto individual plates and observed hourly for the time of the first appearance of eggs. Reported α-times were the interval of time that had passed from the midpoint time of the egg lay until the observation of the first eggs, averaged from a minimum of 20 individual worms.

### Fertility assay

The average self-fertility of each strain was determined by scoring the number of hatched offspring from individual animals. Specifically, 50 gravid adults were allowed to lay eggs for 1 h and were then removed from the plate. Two days later, 20 of the resulting fourth larval stage animals were separated onto individual plates. These were moved onto fresh plates every day until egg laying ceased. Two days after egg laying animals were removed, the plates were scored for the number of worms present. The fertility number reported was the average from 20 scored individual worms.

### Lifespan assays

Synchronous populations were obtained by 1–3 h egg lays with first-day adults, after which the adults were removed and the eggs were left to develop into adults. For strains with a significant incidence of males, late larval stage worms were moved a day before the egg lay, to insure they were unmated. On their first day of adulthood, the synchronous population was moved onto 35 mm NGM assay plates (3 ml NGM agar with 51 μM FUdR to inhibit reproduction). For each condition tested in the lifespan assay, 35–40 worms were placed on each of at least 3 individual plates (referred to as technical replicates) and was considered a single trial (biological replicate).

### Chemical preparation and drugging procedures

Chemical solutions were prepared by dissolving solid chemicals in either sterile water or dimethylsulfoxide (DMSO). For chemicals with water as the solvent, stock solutions were also the working solutions. To drug assay plates, 125 μl of the working solution was added to the top of a 3 ml assay plate. For chemicals with DMSO as the solvent, working solutions were made up from stock solutions and contained 7.5 μl of stock solution for every 125 μl of sterile water. Working solution (132.5 μl) was then added to the top of the plate (final DMSO concentration of 0.25%). Chemicals that precipitated at working solution concentrations were prepared individually from stock solutions (DMSO as solvent) for each plate. Specifically, for each assay plate 7.5 μl of stock solution was placed into a 250 μl tube, to which 125 μl of sterile water was added, the resulting slurry was then dispensed to an assay plate. Chemical solutions were added to plates already containing a ‘lawn' of bacteria in stationary phase. Solutions were distributed over the entire plate surface and allowed to dry in a sterile hood until the surface was devoid of liquid. These were then allowed to sit for 24 h at 20 °C before use or before moving into 4 °C storage for up to 3 weeks.

### Data center

Data in the form of observations was generally recorded during scoring with tablet computers running Filemaker Go (Filemaker Inc.) and was thereby entered into a consolidated Filemaker Database (our Data Center) through a dedicated physical server hosted at the Buck Institute. Unique account IDs were used to facilitate all sites ability to access the entire data, while simultaneously restricting the editing abilities of each site to the data they deposited into the database.

### Statistical analysis

Our overall goals for the analysis of longevity were to (1) partition variation among a wide variety of possible causal sources (for example, genetic differences and experimental ‘error') and (2) test for the effects of compound interventions on individual longevity. These goals required a mix-model approach in which ‘variance-generating' factors were treated as random effects and compounds were treated as fixed effects. To accomplish this we analysed longevity using both GLMs using the *lme4* v.1.12 package and a mixed-model Cox proportional hazard (CPH) model^56^ using the *coxme* v.2.2-5 package^57^ in the R statistical language^58^. Individuals that were lost from the experiment and not directly observed to be dead on a given date were marked as ‘censored'. Censored individuals were retained within the full likelihood framework of the CPH but were excluded from GLM analyses. As we observed a significant association between mean lifespan and its variance across replicate plates (linear regression *R*^2^=0.19, F_1,727_=170.58, *P*<0.0001), we log-transformed the age-at-death data after excluding any censored values for any GLM analysis involving multiple strains. This transformation greatly increased homoscedasticity and reduced the association between mean and variance in longevity (linear regression *R*^2^=0.01, F_1,727_=8.37, *P*=0.0039). Longevities within trials are approximately normal, but frequently not precisely so. However, analysis of variance approaches tend to be robust to departures from normality, especially when homoscedasticity can be achieved^59^. We also analysed the global variance component models using a variety of other error distributions (for example, Poisson; see Supplementary Softwares 1 and 2 for the R-scripts and Supplementary Data 11 and 12 for the outputs from the full statistical analyses). We also provide the results for the analysis on the untransformed scale in the Supplementary Tables. When measured as a relative fraction of the total estimated variance provided for a given distribution, none of the variance component estimates were materially affected by any of these different analyses and all results are qualitatively similar and in close quantitative agreement.

For the analysis of data in the absence of a compound intervention (‘baseline treatments'), laboratory, species, strain, experimenter, trial and plate were taken as the main effects, with strain nested within species. Possible interactions between laboratory–species and laboratory–strain were also included in the model. The other factors were treated as a randomized-block design, with experimenter nested within lab, trial nested within experimenter and plate nested within trial. As we sought to partition the total variance for lifespan among all potential sources, for the baseline treatments all factors were treated as random effects and their associated variance components were estimated using a restricted maximum likelihood approach using *lme4*. Current implementations of the mixed-model CPH do not allow complex models involving interactions among different sets of nested factors and thus that approach could not be used in any of our multi-strain analyses.

For the analysis of lifespans involving compound treatments, the above modelling approach was used with compound as an additional fixed factor in each model. Interactions involving compound and species, strain and laboratory (that is, factors not part of the randomized block design) were also included as random effects in the model. For the global partitioning of variation, we again used a restricted maximum likelihood GLM to partition total variation among all possible non-fixed sources. To test for the effects of individual compounds, we used CPH analysis within each strain so that each compound treatment replicate could be matched with their appropriate replicate-specific control in the randomized blocks design. The CPH analysis was supported by GLM analysis of the same model. Here, the random effects model included lab, experimenter within lab, trial within experimenter and plate within trial for both the GLM and CPH. Compound effects were tested as a planned comparison between the responses of individuals raised on the compound in question and those raised on the appropriate carrier control (H_2_O or DMSO). Results from the two methods of analysis were entirely concordant with one another; thus, we present a summary of CPH results in the figures and the full results for both analyses in Supplementary Tables 3 and 4. Using our per-strain estimates of sampling variance, we expect to have power >0.9 to detect a compound effect of at least 10% within each trial within each lab.

For the analysis of the timing of reproductive development (α-time), we found a strong relationship between the mean and variance of time to first egg lay (measured in hours) among replicates (linear regression *R*^2^=0.17, F_1,119_=23.42, *P*<0.0001). Analysing this trait as a developmental rate (1/α-time) stabilized the variances and eliminated this relationship (linear regression *R*^2^=0.01, F_1,119_=1.08, *P*=0.3009), and we therefore used developmental rate as the focal trait for our analysis of variance. We detected no significant relationship between mean and variance for lifetime fertility among replicates (linear regression *R*^2^=0.01, *F*_1,98_=1.08, *P*<0.3012), and so that trait was analysed on the original scale. As with longevity, we partitioned potential sources of variation for developmental rate and fertility using a GLM including the same causal factors. Unlike longevity, however, there is no plate effect in the model because each individual was raised on their own plate. Further, these assays were not as extensively replicated as the longevity trials; thus, the effects of individual experimenters are subsumed into the general laboratory factor in the partitioning of variation. *C. elegans* strain QX1211 displayed extraordinarily low levels of fertility, probably as a result of the ‘mortal germline' phenotype^60^ and thus was excluded from the variance component analysis for this trait.

### Data availability

Statistical summaries (Supplementary Data 1–5), raw data (Supplementary Data 6–10), R codes (Supplementary Software 1 and 2) and analysis (Supplementary Data 11 and 12) are provided as Supplementary Materials. Detailed protocols for the experiments described here have been published at Protocol Exchange^41^.

## Additional information

**How to cite this article:** Lucanic, M. *et al*. Impact of genetic background and experimental reproducibility on identifying chemical compounds with robust longevity effects. *Nat. Commun.* **8,** 14256 doi: 10.1038/ncomms14256 (2017).

**Publisher's note**: Springer Nature remains neutral with regard to jurisdictional claims in published maps and institutional affiliations.

## Supplementary Material

Supplementary Information Supplementary Figures, Supplementary Tables.

Supplementary Data 1 Statistical summaries from development dataset

Supplementary Data 2 Statistical summaries from fertility dataset

Supplementary Data 3 Statistical summaries from lifespan (no chemicals) dataset

Supplementary Data 4 Statistical summaries from lifespan (chemicals) dataset

Supplementary Data 5 Statistical summaries from lifespan (dose response) dataset

Supplementary Data 6 Raw development dataset

Supplementary Data 7 Raw fertility dataset

Supplementary Data 8 Raw lifespan (no chemicals) dataset

Supplementary Data 9 Raw lifespan (chemicals) dataset

Supplementary Data 10 Raw lifespan (dose response) dataset

Supplementary Data 11 Raw output from analysis of lifespan (no chemicals) dataset

Supplementary Data 12 Raw output from analysis of lifespan (chemicals) dataset

Supplementary Software 1 VarComps.R: This R package computes the variance component estimates for reproducibility across each of the datasets using hierarchical nested general linear models under a variety of possible error distributions.

Supplementary Software 2 CompoundTests.R: This R package computes the effects of compounds on longevity using both general linear models and Cox Proportional-Hazards models with random effects.

## Figures and Tables

**Table 1:** Variation attributable to different sources.

**Source of variation**	**Developmental rate**	**Fertility**
Genetic variation	**83.1**	**63.3**
Among species	77.8*	50.9*
Among strains w/in species	5.3*	12.4*
Reproducibility among labs	**8.3**	**7.9**
Among labs	0.0	1.4
Lab × species	4.2*	0.0
Lab × strain	4.1*	6.5*
Reproducibility within labs	**3.8**	**5.6**
Among trials w/in lab	3.8*	5.6*
Individual variation	**4.8**	**23.3**
Total	**100**	**100**
Total number of observations	1,887	1,667

Variation attributable to different sources within the hierarchical analysis of reproducibility within and among labs, species and strains in the absence of any chemical interventions for developmental rate and fertility. Each entry represents the percentage of the total trait variance explained by that particular factor as estimated by a general linear model. Larger values mean more variability attributable to that source, whereas lower values imply more consistent results across that source. Bold entries indicate a summation of the component numbers immediately beneath it.

^*^95% Confidence interval bounded from zero in the maximum likelihood procedure (see Supplementary Table 1).

**Table 2:** Reproducibility of longevity estimates within and between labs.

**Source of variation**	**Baseline longevity**	**Compound longevity**
Genetic variation	**19.7**	**44.9**
Among species	11.7*	30
Among strains w/in species	8.0*	8.6*
Species × compound		3.5*
Strain × compound		2.8*
Reproducibility among labs	**7.5**	**1**
Among labs	0.0	0.0
Lab × species	0.6	0.1
Lab × strain	6.9*	0.5*
Lab × compound		0.4*
Reproducibility within labs	**15.4**	**9.7**
Among experimenters w/in lab	0.0	1.6*
Among trials w/in lab	9.3*	1.8*
Among plates w/in trials	6.1*	6.3*
Individual variation	**57.4**	**44.4**
Total	**100**	**100**
Total number of observations	26,333	44,919

Reproducibility of longevity estimates within and between labs for the baseline analysis of 22 strains across three species with no added compounds and tests of pharmacological intervention for 10 different compounds for six strains across two species. Variance estimates for the compound trials are the averages across all compounds as estimated from a single general linear model. Bold entries indicate a summation of the component numbers immediately beneath it.

^*^95% Confidence interval bounded from zero in the maximum likelihood procedure (see Supplementary Table 2).
