# Supplementary material for: Impact of genetic background and experimental reproducibility on identifying chemical compounds with robust longevity effects
Source: Nat Commun. 2017 Feb 21;8:14256. doi: 10.1038/ncomms14256 (PMC5321775; doi:10.1038/ncomms14256)
Supplement: Supplementary Information — Supplementary Figures, Supplementary Tables. [file ncomms14256-s1.pdf]

Supplementary Information (Figures 1-8 and Tables 1-4):

Supplementary Figure 1:

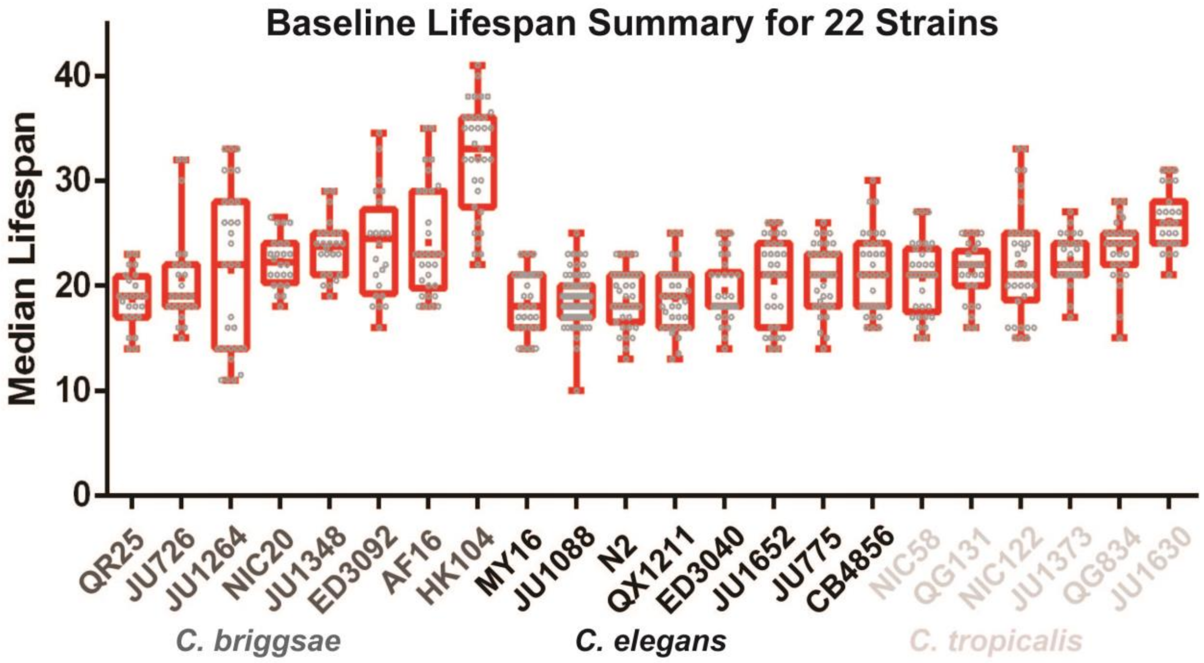

Supplementary Figure 1: Summary of median lifespans for 22 *Caenorhabditis* strains

Graphical summary of the median lifespans of 22 *Caenorhabditis* strains. Median lifespans shown represent single plate populations (single plate lifespan assays starting with 35-40 animals) assessed at 3 geographically distinct sites. For this graph, censored values were not included in the median lifespan calculations. Graph is segregated by species, such that 8 *C. briggsae* strains are indicated in gray text, 8 *C. elegans* strains are shown in black text and 6 *C. tropicalis* strains are shown in off white text. Each plotted value is shown in grey, with red 'box and whisker' plots overlaid. Red boxes encompass the 25th to 75th percentiles of the values and whiskers denote the minimum to maximum. Red bar inside the box labels the median of the plotted values, while the red dot marks the mean. Statistical analysis of the parent data used to generate this graph is presented in table 1, while lifespan curves are shown in main text figures 2-3. Sample sizes and summary statistics for individual replicates are provided in Supplementary Table 7.

Supplementary Figure 2:

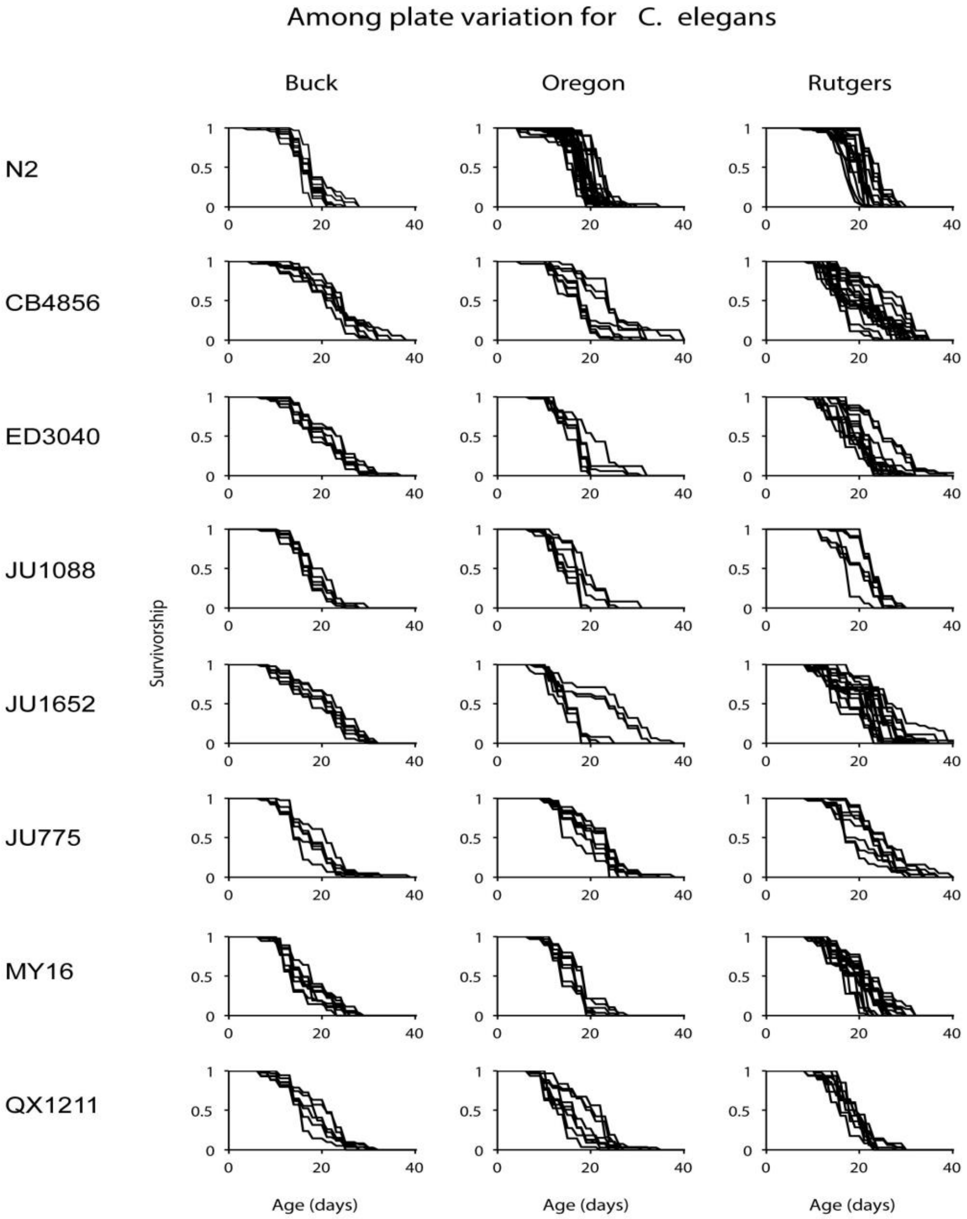

Supplementary Figure 2: Variation in longevity within labs for each replicate plate for laboratory and natural isolates of *C. elegans*

Graphs show the survivorship curves for each plate population tested for all of the *C. elegans* strains assessed. Curves are segregated into panels according to the strain and site where the assay was performed. Each plate was initiated with n = 35 animals.

Supplementary Figure 3:

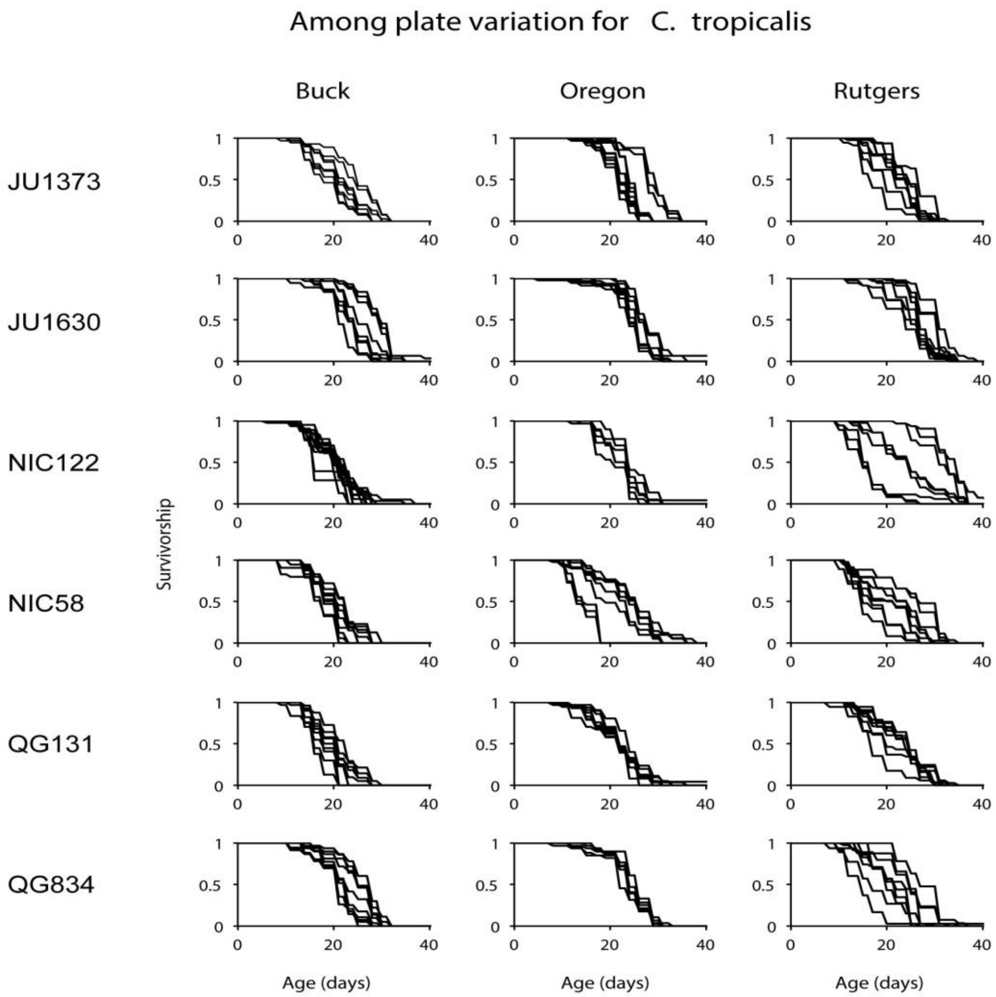

Supplementary Figure 3: Variation in longevity within labs for each replicate plate for six natural isolates of *C. tropicalis*

Graphs show the survivorship curves for each plate population tested for all of the *C. tropicalis* strains assessed. Curves are segregated into panels according to the strain and site where the assay was performed. Each plate was initiated with n = 35 animals.

Supplementary Figure 4:

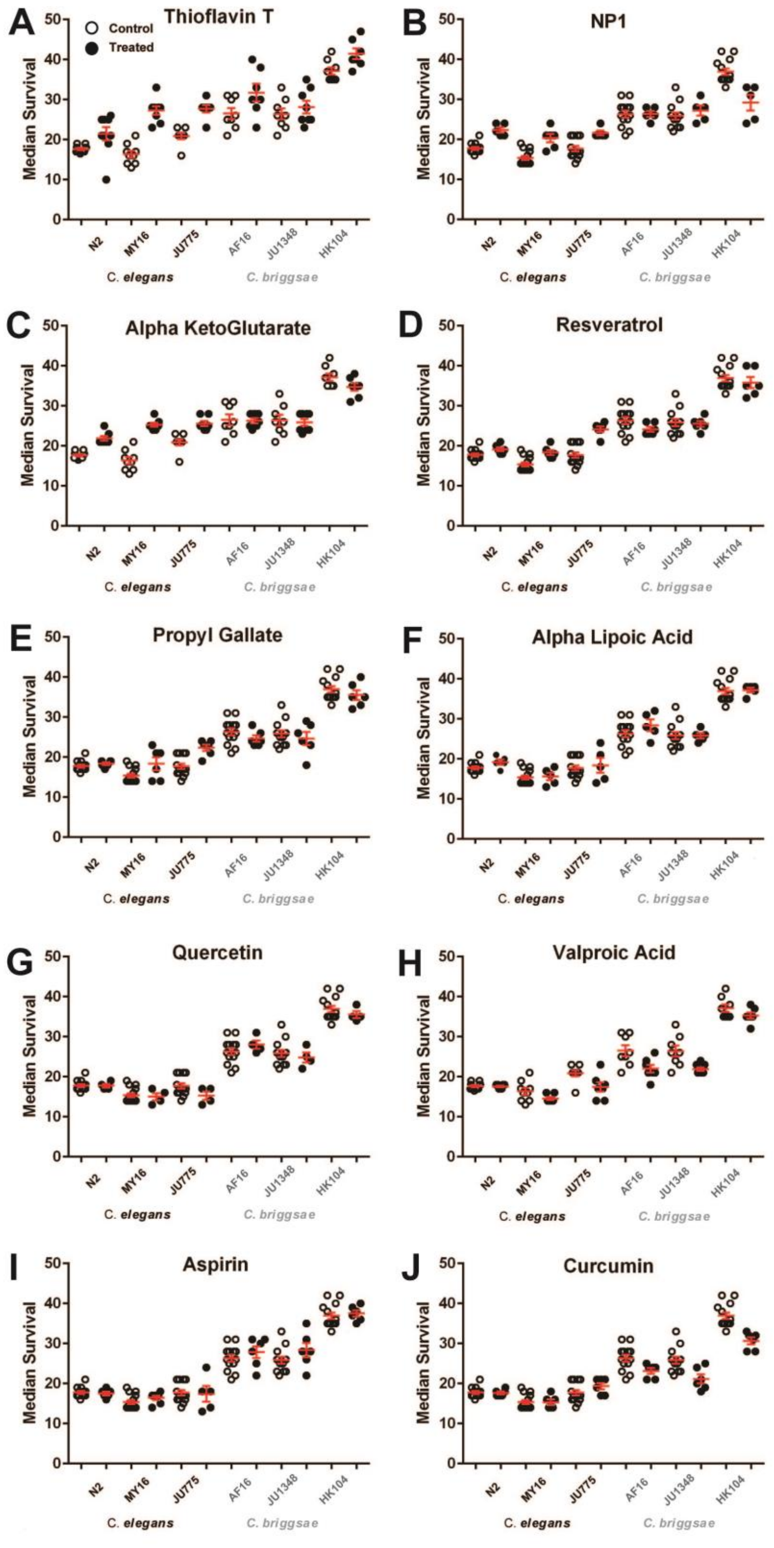

Supplementary Figure 4: Summary of median lifespans for chemical and control lifespan assays

Each panel (A-J) presents a graph of the observed median lifespans of 6 *Caenorhabditis* strains treated chronically from the first day of adulthood with either solvent control (H_20 or DMSO) or a specific chemical (chemical is defined above each graph). Data is presented in a 'by trial' format (each trial consisted of 3 replicate plates of a generational cohort, initiated with a total of 105-120 animals). In this figure control values are over represented relative to individual compounds since every control trial is shown for each chemical that used that control. As multiple chemicals used the same type control (particularly DMSO controls), more control experiments were performed than any given chemical. Points are color coded to indicate whether values are from control or chemical treated animals, as indicated in legend on panel (A). Censored animals were not included when calculating the median lifespans shown in this graph, which can result in underestimation of the median lifespan. Large red bars represent the mean with small red bars indicating the standard error. Summaries from the statistical analysis of the parent data used to generate these graphs is included in table 2 and supplementary tables 2-4. Sample sizes and summary statistics for individual replicates are provided in Supplementary Table 8.

Supplementary Figure 5:

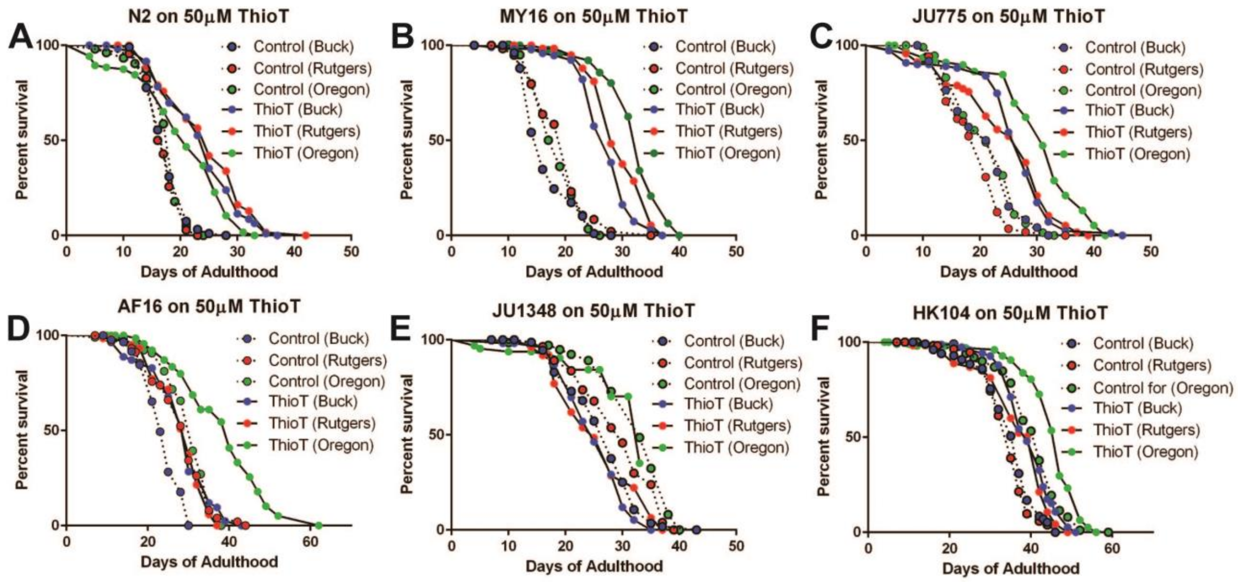

Supplementary Figure 5: Representative survivorship curves from control and Thioflavin T treated generational cohorts

Each panel (A-F) shows representative survivorship plots for a *Caenorhabditis* strain (indicated above panel) treated chronically from the first day of adulthood with either solvent control (H_20) or Thioflavin T (ThioT). Data is presented in a 'by trial' format (each trial consisted of 3 replicate plates initiated with a total of 105-120 animals). For each strain, 1 trial is shown from each research site (color coded and described in each panel key) consisting of both the treated animals (solid lines) and their specific matched controls (same generational cohort scored in parallel, dotted lines). This data was also included in figure 4 and summaries from the statistical analysis of the parent data used to generate these graphs is included in table 2 and supplementary tables 2-4. Sample sizes and summary statistics for individual replicates are provided in Supplementary Table 8.

Supplementary Figure 6:

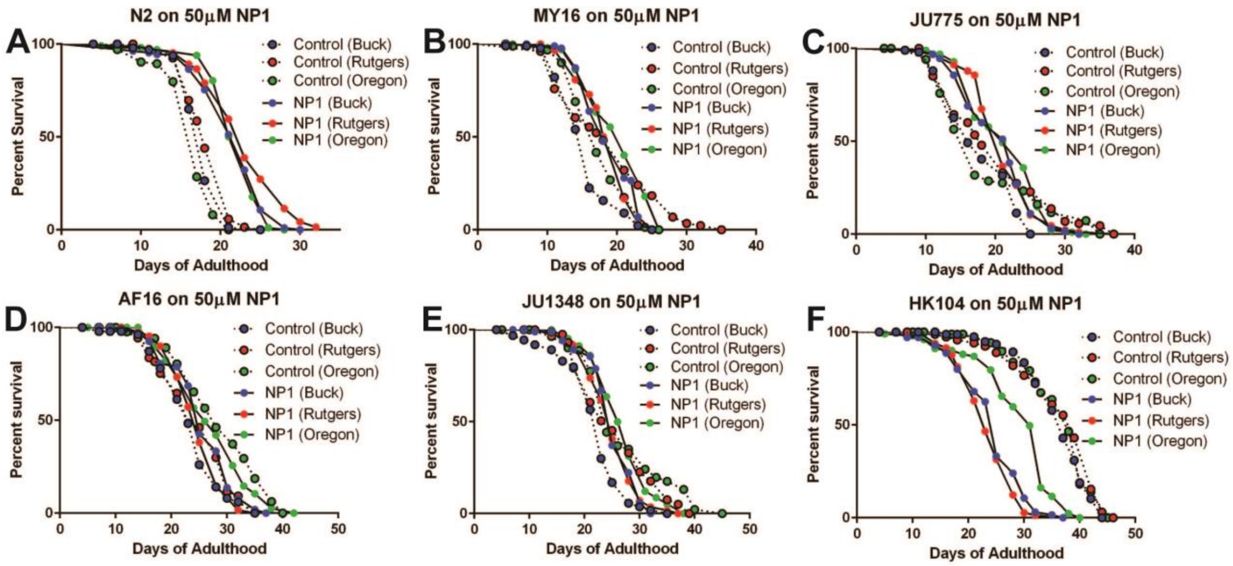

Supplementary Figure 6: Representative survivorship curves from control and NP1 treated generational cohorts

Each panel (A-F) shows representative survivorship plots for a *Caenorhabditis* strain (indicated above panel) treated chronically from the first day of adulthood with either solvent control (DMSO) or NP1. Data is presented in a 'by trial' format (each trial consisted of 3 replicate plates initiated with a total of 105-120 animals). For each strain, 1 trial is shown from each research site (color coded and described in each panel key) consisting of both the treated animals (solid lines) and their specific matched controls (same generational cohort scored in parallel, dotted lines). Sample sizes and summary statistics for individual replicates are provided in Supplementary Table 8.

Supplementary Figure 7:

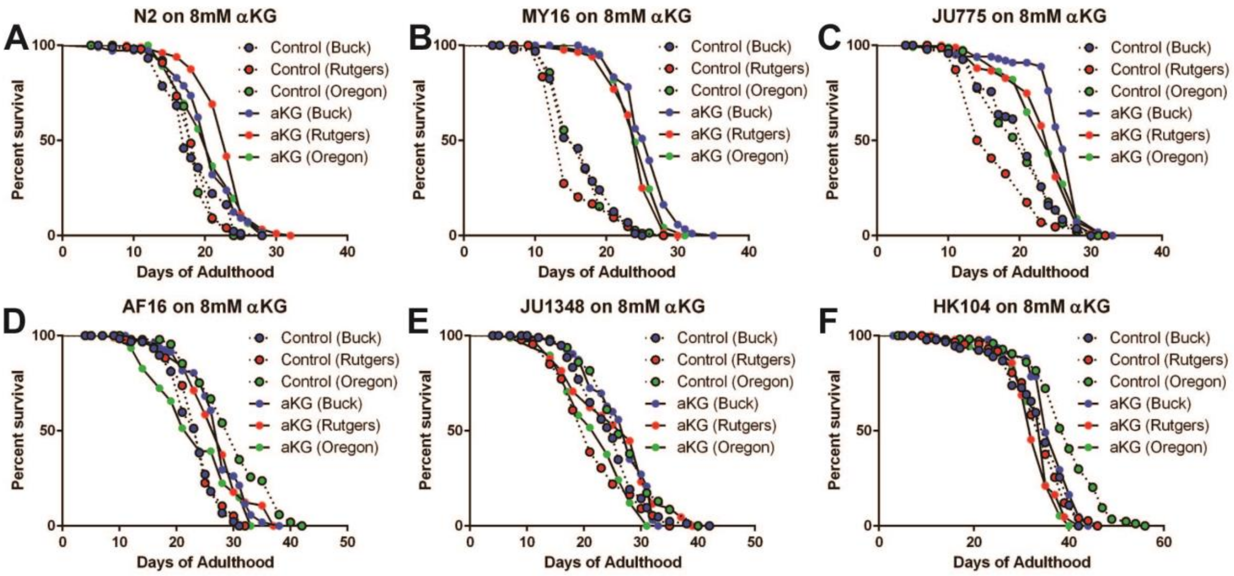

Supplementary Figure 7: Representative survivorship curves from control and αKetoglutarate treated generational cohorts

Each panel (A-F) shows representative survivorship plots for a *Caenorhabditis* strain (indicated above panel) treated chronically from the first day of adulthood with either solvent control (H_20) or αKetoglutarate (αKG). Data is presented in a 'by trial' format (each trial consisted of 3 replicate plates initiated with a total of 105-120 animals). For each strain, 1 trial is shown from each research site (color coded and described in each panel key) consisting of both the treated animals (solid lines) and their specific matched controls (same generational cohort scored in parallel, dotted lines). Sample sizes and summary statistics for individual replicates are provided in Supplementary Table 8.

Supplementary Figure 8:

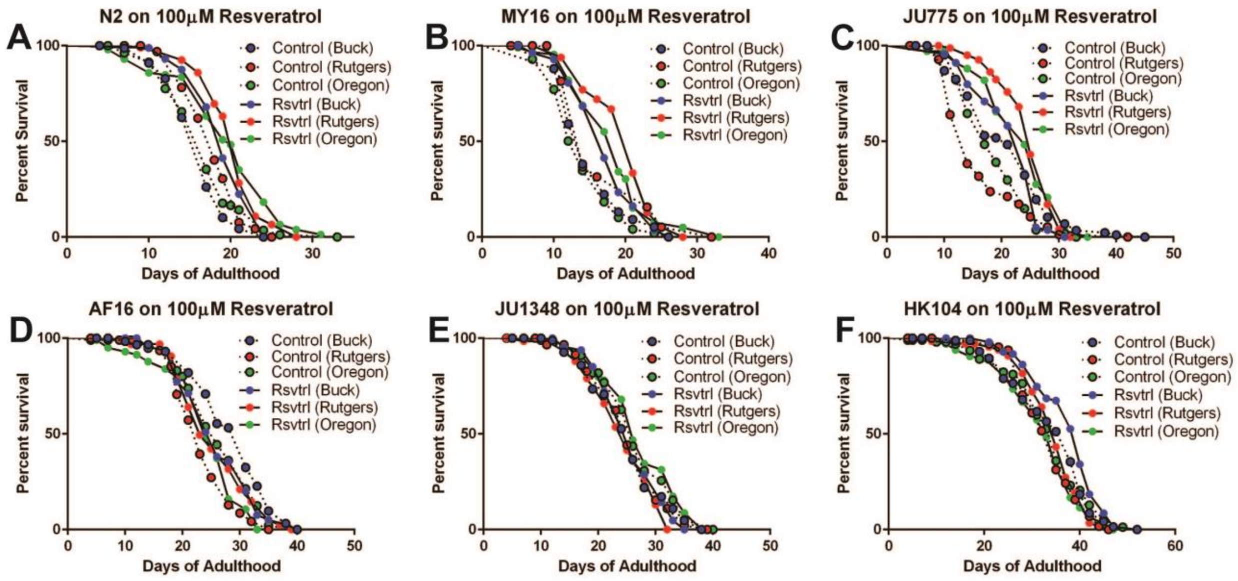

Supplementary Figure 8: Representative survivorship curves from control and Resveratrol treated generational cohorts

Each panel (A-F) shows representative survivorship plots for a *Caenorhabditis* strain (indicated above panel) treated chronically from the first day of adulthood with either solvent control (DMSO) or Resveratrol (rsvtrl). Data is presented in a 'by trial' format (each trial consisted of 3 replicate plates initiated with a total of 105-120 animals). For each strain, 1 trial is shown from each research site (color coded and described in each panel key) consisting of both the treated animals (solid lines) and their specific matched controls (same generational cohort scored in parallel, dotted lines). Sample sizes and summary statistics for individual replicates are provided in Supplementary Table 8.

Supplementary Table 1. Partitioning of variation for developmental rate and fertility across genetic background and experimental replicates within and among labs. Variance components were estimated as a randomized block design using a restricted maximum likelihood (REML) general linear model using the *lme4* package in R. All factors were treated as random effects.

A. Variance component estimates for developmental rate (i.e., the inverse of the age at first reproduction). Sample sizes and summary statistics are presented in Supplementary Table 5.

Source	Variance Component	Lower 95% CI	Upper 95% CI	Percent of Total
Species	1.70E-06	2.68E-07	1.01517E-05	77.8
Strain	1.16E-07	4.77E-08	2.71E-07	5.3
Lab	0.0	0	2.82E-07	0.0
Species*Lab	9.04E-08	1.45E-08	4.52E-07	4.2
Strain*Lab	8.88E-08	5.51E-08	1.51E-07	4.1
Trial[Lab]	8.34E-08	4.53E-08	1.68E-07	3.8
Residual	1.06E-07	9.89E-08	1.13E-07	4.8
Total	2.18E-06			100.0

B. Variance component estimates for lifetime fertility. Estimates for the GLM with normally distributed errors are given in the left columns and those from a GLM with Poisson distributed errors are given in the right columns. Sample sizes and summary statistics are presented in Supplementary Table 6.

Source	Variance Component (Normal)	Lower 95% CI	Upper 95% CI	Percent of Total	Variance Component (Poisson-sqrt)	Lower 95% CI	Upper 95% CI
Species	4361	635	26803	50.9	4.27	0.97	39.67
Strain	1058	456	2481	12.3	1.46	0.62	3.4
Lab	115	0	2600	1.4	0.09	0	2.80
Species*Lab	0	0	397	0.0	0	0	0.49
Strain*Lab	560	324	995	6.5	0.94	0.61	1.57
Trial[Lab]	476	245	983	5.6	0.61	0.35	1.20
Residual	1992	1859	2137	23.26			
Total	8746	3829	36059	100.0			

Supplementary Table 2. Partitioning of variation for longevity across genetic background and experimental replicates within and among labs. Variance components were estimated as a randomized block design using a restricted maximum likelihood (REML) general linear model using the *lme4* package of R. All factors were treated as random effects except for “compound” in Table S1B. Tests involving the fixed compound effects are reported in Supplementary Table 4.

A. Variance component estimates for the natural log of individual lifespan from the 22-strain, 3-species baseline longevity experiments. Sample sizes and summary statistics are presented in Supplementary Table 7.

Source	Variance Component	Lower 95% CI	Upper 95% CI	Percent of Total
Species	1.22E-02	5.37E-04	8.12E-02	11.7
Strain	8.41E-03	3.22E-03	2.00E-02	8.0
Lab	0.0	0.0	9.90E-03	0.0
Species*Lab	5.85E-04	0.0	7.46E-03	0.6
Strain*Lab	7.21E-03	4.32E-03	1.25E-02	6.9
Experimenter[Lab]	0.0	0.0	3.53E-03	0.0
Trial[Lab, Experimenter]	9.74E-03	6.41E-03	1.52E-02	9.3
Plate[Lab, Experimenter, Trial]	6.42E-03	5.51E-03	7.48E-03	6.1
Residual	6.00E-02	5.89E-02	6.12E-02	57.4
Total	1.05E-01			100.0

B. Average variance component estimates for the natural log of individual lifespan from the six-strain, ten-compound experiments. See Supplementary Table 3 for a per-strain analysis of replication. Sample sizes and summary statistics are presented in Supplementary Table 8.

Source	Variance Component	Lower 95% CI	Upper 95% CI	Percent of Total
Species	5.31E-02	0.0	5.15E-01	30.00
Species*Compound	6.18E-03	2.96E-04	6.42E-02	3.49
Strain[Species]	1.53E-02	4.38E-03	9.56E-02	8.63
Strain*Compound[Species]	5.01E-03	3.10E-03	8.34E-03	2.83
Lab	0.0	0.0	1.17E-02	0.00
Lab*Compound	6.85E-04	9.19E-05	1.64E-03	0.39
Lab*Species	2.00E-04	0.0	4.75E-03	0.11
Lab*Strain[Species]	8.46E-04	0.0	2.98E-03	0.48
Experimenter[Lab]	2.80E-03	0.0	8.88E-03	1.58
Trial[Lab,Experimenter]	3.10E-03	1.69E-03	5.93E-03	1.75
Plate[Lab,Experimenter,Trial]	1.12E-02	1.00E-02	1.26E-02	6.34
Residual	7.86E-02	7.74E-02	7.99E-02	44.40
Total	1.77E-01			100.00

Supplementary Table 3. Variance components estimates for longevity for the 10-compound experiments, analyzed separately for each strain. Values are from a hierarchical randomized block design estimated either via a restricted maximum likelihood general linear model using the *lme4* package (v. 1.1-12) or via a random effects Cox Proportional Hazards model as implemented by the *coxme* package (v. 2.2-5) in R (Therneau 2012).

A. ***C. elegans*** **N2** (*n* = 7,962)

	General Linear Model				Cox Prop Hazard
Source	Var Comp	Lower 95% CI	Upper 95% CI	Percent Total	Var Comp
Lab	0.88	0.07	5.79	4.4	0.03
Experimenter[Lab]	0.14	0	1.39	0.7	0.01
Trial[Lab,Expter]	0.67	0.08	1.51	3.3	0.03
Plate[Lab,Expter,Trial]	2.13	1.49	2.63	10.7	0.10
Residual	16.18	15.6	16.7	80.9	
Total	20.00			100.0	

B. ***C. elegans*** **MY16** (*n* = 7,284)

	General Linear Model				Cox Prop Hazard
Source	Var Comp	Lower 95% CI	Upper 95% CI	Percent Total	Var Comp
Lab	0.25	0.0	2.14	1.2	0.02
Experimenter[Lab]	0.0	0.0	1.30	0.0	0.0
Trial[Lab,Expter]	1.73	0.89	2.93	8.3	0.10
Plate[Lab,Expter,Trial]	1.53	0.98	1.93	7.33	0.05
Residual	17.30	16.64	17.97	83.1	
Total	20.79			100.0	

C. ***C. elegans*** **JU775** (*n* = 7,245)

	General Linear Model				Cox Prop Hazard
Source	Var Comp	Lower 95% CI	Upper 95% CI	Percent Total	Var Comp
Lab	0.0	0.0	0.80	0.0	0.03
Experimenter[Lab]	0.18	0.0	1.78	0.5	0.01
Trial[Lab,Expter]	2.24	0.98	4.05	6.0	0.06
Plate[Lab,Expter,Trial]	2.48	1.57	3.16	6.6	0.05
Residual	32.57	31.40	33.81	86.9	
Total	37.47			100.0	

D. ***C. briggsae*** **AF16** (*n* = 7,459)

Source	General Linear Model				Cox Prop Hazard
	Var Comp	Lower 95% CI	Upper 95% CI	Percent Total	Var Comp
Lab	0.78	0.0	5.83	1.6	0.26
Experimenter[Lab]	0.50	0.0	3.04	1.1	0.09
Trial[Lab,Expter]	0.36	0.0	1.96	0.8	0.02
Plate[Lab,Expter,Trial]	5.53	3.44	7.08	11.5	0.36
Residual	40.77	38.96	42.71	85.0	
Total	47.90			100.0	

E. ***C. briggsae*** **HK104** (*n* = 7,340)

Source	General Linear Model				Cox Prop Hazard
	Var Comp	Lower 95% CI	Upper 95% CI	Percent Total	Var Comp
Lab	0.0	0.0	6.82	0.0	0.06
Experimenter[Lab]	5.71	0.0	18.34	7.5	0.00
Trial[Lab,Expter]	7.61	3.78	14.70	10.0	0.09
Plate[Lab,Expter,Trial]	7.02	4.73	8.74	11.5	0.20
Residual	55.69	53.58	57.93	73.2	
Total	76.95			100.0	

F. ***C. briggsae*** **JU1348** (*n* = 7,629)

Source	General Linear Model				Cox Prop Hazard
	Var Comp	Lower 95% CI	Upper 95% CI	Percent Total	Var Comp
Lab	0.0	0.0	0.65	0.0	0.03
Experimenter[Lab]	0.0	0.0	0.83	0.0	0.01
Trial[Lab,Expter]	2.22	0.89	4.16	5.0	0.06
Plate[Lab,Expter,Trial]	5.02	3.24	6.31	11.3	0.13
Residual	37.07	35.51	38.74	83.7	
Total	44.53			100.0	

Supplementary Table 4. Significance tests for compound interventions effects on longevity. Each effect is tested using both a general linear model of age at death and random effects Cox Proportional Hazard Model. Each compound is tested as a planned comparison against its appropriate carrier control. Variance components estimates for the randomized-block effects that were included in the overall model are presented in Supplementary Table 3.

A. *C. elegans* N2

	General linear model				Random effects Cox Proportional Hazard			
Compound	Effect	Stderr	z-value	p-value	Effect	Stderr	z-value	p-value
Alpha ketoglutarate	3.78	0.51	7.42	<1E-05	1.06	0.11	9.33	<1E-05
Alpha lipoic acid	1.49	0.54	2.74	0.0401	0.44	0.12	3.67	0.0017
Aspirin	0.25	0.51	0.49	0.9983	0.03	0.11	0.23	1.0
Curcumin	0.04	0.50	0.08	1.0	-0.07	0.11	-0.66	0.9898
NP1	4.11	0.53	7.69	<1E-04	1.10	0.12	9.43	<1E-04
Propyl gallate	1.79	0.50	3.59	0.0023	0.55	0.11	4.98	<1E-04
Quericitin	0.51	0.56	0.91	0.9422	0.12	0.13	0.95	0.9286
Resveratrol	1.93	0.50	3.84	0.0008	0.71	0.11	6.41	<1E-04
Thioflavin T	3.26	0.38	8.55	<1E-05	1.49	0.09	16.46	<1E-05
Valproic acid	-0.37	0.51	-0.72	0.8330	-0.06	0.12	-0.52	0.9290

B. *C. elegans* MY16

	General linear model				Random effects Cox Proportional Hazard			
Compound	Effect	Stderr	z-value	p-value	Effect	Stderr	z-value	p-value
Alpha ketoglutarate	8.01	0.50	16.03	<1E-06	1.66	0.10	16.08	<1E-10
Alpha lipoic acid	-0.20	0.55	0.35	0.9998	0.02	0.12	0.20	1.0
Aspirin	0.41	0.49	0.85	0.9607	0.05	0.10	0.51	0.9980
Curcumin	-0.47	0.50	-0.93	0.9371	-0.25	0.10	-2.39	0.106
NP1	3.65	0.49	7.47	<1E-05	0.86	0.10	8.54	<1E-04
Propyl gallate	2.21	0.50	4.41	<1E-04	0.55	0.10	5.35	<1E-04
Quericitin	-0.22	0.55	-0.39	0.9996	-0.10	0.11	-0.84	0.9630
Resveratrol	2.13	0.50	4.24	0.0002	0.52	0.10	4.93	<1E-04
Thioflavin T	9.97	0.45	22.05	<1E-06	2.40	0.10	25.00	<1E-10
Valproic acid	-2.48	0.50	-4.95	<1E-05	-0.82	0.10	-7.99	<1E-10

C. *C. elegans* JU775

	General linear model				Random effects Cox Proportional Hazard			
Compound	Effect	Stderr	z-value	p-value	Effect	Stderr	z-value	p-value
Alpha ketoglutarate	4.77	0.66	7.25	<0.001	0.77	0.10	7.53	<1E-04
Alpha lipoic acid	-0.53	0.68	-0.79	0.9735	-0.22	0.11	-2.09	0.2112
Aspirin	-0.33	0.62	-0.53	0.9973	-0.09	0.10	-0.88	0.9526
Curcumin	0.71	0.62	1.14	0.8411	-0.01	0.10	-0.15	1.0
NP1	3.45	0.62	5.54	<1E-04	0.48	0.10	5.00	<1E-04
Propyl gallate	2.27	0.62	3.67	0.0005	0.39	0.10	4.01	0.0004
Quericitin	-0.74	0.67	-1.11	0.8617	-0.14	0.10	-1.30	0.7387
Resveratrol	3.97	0.63	6.30	<1E-04	0.56	0.10	5.71	<1E-04
Thioflavin T	5.20	0.65	7.98	<0.001	1.12	0.10	11.16	<1E-04
Valproic acid	-1.89	0.64	-2.93	0.0094	-0.39	0.10	-3.97	0.0002

D. *C. briggsae* AF16

	General linear model				Random effects Cox Proportional Hazard			
Compound	Effect	Stderr	z-value	p-value	Effect	Stderr	z-value	p-value
Alpha ketoglutarate	-1.08	0.94	-1.15	0.5370	0.03	0.15	0.23	0.9920
Alpha lipoic acid	1.06	0.92	1.15	0.8320	0.14	0.14	1.01	0.9020
Aspirin	-0.20	0.87	-0.24	1.0	0.07	0.13	0.50	0.9980
Curcumin	-4.67	0.87	-5.37	<1E-04	-0.72	0.13	-5.44	<1E-04
NP1	-0.09	0.88	-0.11	1.0	-0.04	0.13	-0.26	1.0
Propyl gallate	-1.33	0.83	-1.61	0.5040	0.13	0.13	-1.30	0.7350
Quericitin	0.38	0.97	0.39	1.0	0.12	0.15	0.78	0.9730
Resveratrol	-1.89	0.85	-2.21	0.1580	-0.17	0.13	-1.32	0.7180
Thioflavin T	4.45	0.95	4.70	<1E-04	0.92	0.14	6.43	<1E-06
Valproic acid	-4.83	1.00	-4.86	<1E-04	-0.81	0.15	-5.26	<1E-06

E. *C. briggsae* HK104

	General linear model				Random effects Cox Proportional Hazard			
Compound	Effect	Stderr	z-value	p-value	Effect	Stderr	z-value	p-value
Alpha ketoglutarate	-3.17	1.03	-3.09	0.0056	-0.63	0.17	-3.83	<0.001
Alpha lipoic acid	0.07	1.09	0.07	1.0	-0.02	0.17	-0.13	1.0
Aspirin	0.52	0.99	0.52	1.0	0.13	0.16	0.80	0.9705
Curcumin	-3.53	1.12	-3.55	0.0026	-0.75	0.16	-4.81	<1E-04
NP1	-8.63	1.00	8.64	<1E-4	-1.53	0.15	-10.05	<1E-04
Propyl gallate	-1.41	1.00	1.42	0.6517	-0.22	0.16	-1.38	0.6795
Quericitin	-3.45	1.12	-3.08	0.0139	-0.66	0.18	-3.69	0.0016
Resveratrol	-1.04	1.01	-1.03	0.9006	-0.18	0.16	-1.08	0.8737
Thioflavin T	3.49	1.02	3.43	0.0018	0.61	0.17	3.69	<0.001
Valproic acid	-3.02	1.02	-2.95	0.0091	-0.46	0.17	-2.79	0.0150

F. *C. briggsae* JU1348

	General linear model				Random effects Cox Proportional Hazard			
Compound	Effect	Stderr	z-value	p-value	Effect	Stderr	z-value	p-value
Alpha ketoglutarate	-2.04	0.88	-2.31	0.0552	-0.31	0.14	-2.14	0.0833
Alpha lipoic acid	1.04	0.93	1.11	0.8560	0.15	0.15	0.96	0.9280
Aspirin	0.73	0.86	0.85	0.9600	0.19	0.14	1.34	0.7100
Curcumin	-4.14	0.84	-4.96	<1E-4	-0.90	0.14	-6.63	<1E-04
NP1	1.96	0.84	2.33	0.1210	0.00	0.14	0.02	1.0
Propyl gallate	-0.91	0.83	1.09	0.8700	-0.17	0.14	-1.25	0.7730
Quericitin	0.42	0.94	0.45	0.9990	-0.13	0.15	-0.85	0.9590
Resveratrol	-0.20	0.86	-0.24	1.0	0.00	0.14	0.00	1.0
Thioflavin T	1.04	0.90	1.16	0.5104	0.21	0.15	1.45	0.3324
Valproic acid	-4.18	0.88	-4.74	<0.001	-0.84	0.14	-5.87	<0.001
